# The Role of IL-13 and IL-4 in Adipose Tissue Fibrosis

**DOI:** 10.3390/ijms24065672

**Published:** 2023-03-16

**Authors:** Lilli Arndt, Andreas Lindhorst, Julia Neugebauer, Anne Hoffmann, Constance Hobusch, Vasileia-Ismini Alexaki, Adhideb Ghosh, Matthias Blüher, Christian Wolfrum, Markus Glaß, Martin Gericke

**Affiliations:** 1 Institute of Anatomy, Leipzig University, 04103 Leipzig, Germany; 2 Institute of Anatomy and Cell Biology, Martin-Luther-University Halle-Wittenberg, 06108 Halle (Saale), Germany; 3 Helmholtz Institute for Metabolic, Obesity and Vascular Research, 04103 Leipzig, Germany; 4 Institute of Clinical Chemistry and Laboratory Medicine, University Hospital Dresden, 01307 Dresden, Germany; 5 Institute of Food, Nutrition and Health, ETH Zurich, 8603 Schwerzenbach, Switzerland; 6 Functional Genomics Center Zurich, ETH Zurich and University of Zurich, 8057 Zurich, Switzerland; 7 Institute of Molecular Medicine, Martin-Luther-University Halle-Wittenberg, 06120 Halle (Saale), Germany

**Keywords:** adipose tissue, fibrosis, IL-13, IL-4, macrophages, obesity

## Abstract

White adipose tissue (WAT) fibrosis, characterized by an excess of extracellular (ECM) matrix components, is strongly associated with WAT inflammation and dysfunction due to obesity. Interleukin (IL)-13 and IL-4 were recently identified as critical mediators in the pathogenesis of fibrotic diseases. However, their role in WAT fibrosis is still ill-defined. We therefore established an ex vivo WAT organotypic culture system and demonstrated an upregulation of fibrosis-related genes and an increase of α-smooth muscle actin (αSMA) and fibronectin abundance upon dose-dependent stimulation with IL-13/IL-4. These fibrotic effects were lost in WAT lacking *il4ra*, which encodes for the underlying receptor controlling this process. Adipose tissue macrophages were found to play a key role in mediating IL-13/IL-4 effects in WAT fibrosis as their depletion through clodronate dramatically decreased the fibrotic phenotype. IL-4-induced WAT fibrosis was partly confirmed in mice injected intraperitoneally with IL-4. Furthermore, gene correlation analyses of human WAT samples revealed a strong positive correlation of fibrosis markers with IL-13/IL-4 receptors, whereas *IL13* and *IL4* correlations failed to confirm this association. In conclusion, IL-13 and IL-4 can induce WAT fibrosis ex vivo and partly in vivo, but their role in human WAT remains to be further elucidated.

## 1. Introduction

Obesity is one of the gravest global health problems in western societies, leading to an increasing risk for cardiovascular and metabolic diseases including dyslipidemia, insulin resistance, and type 2 diabetes [1]. Obesity-related chronic nutrient overload promotes white adipose tissue (WAT) expansion through both increased lipid storage in adipocytes (hypertrophy) and formation of new adipocytes (hyperplasia); these are accompanied by extracellular matrix (ECM) remodeling to accommodate adipose tissue modifications. Further obesity-associated dysregulations in WAT, such as hypoxia and inflammation characterized by accumulation of macrophages and other immune cells, can advance WAT fibrosis [2]. Fibrosis of adipose tissue is considered both a feature of obesity and a contributing factor to loss of WAT function and plasticity [3] and is closely correlated to more metabolic complications [4]. As with other fibrotic diseases, WAT fibrosis arises due to the excessive accumulation of ECM proteins; this can either result from increased production or impaired degradation of ECM components such as collagens I, III, VI, and fibronectin [5], which are primarily produced by adipocyte progenitors, adipocytes, fibroblasts, and myofibroblasts [4]. Immune cells, such as mast cells and adipose tissue macrophages (ATMs), appear to have the greatest regulatory role in ECM production through their effects on other cell types via cytokine secretion [6,7]. It is widely accepted that transforming growth factor β-1 (TGF-β1), interleukin (IL)-4, and IL-13 are the most prominent cytokines driving fibrosis in liver, lung, kidney, and skin [8]. 

IL-4 and IL-13 are the key cytokines in the type 2 immune responses, sharing structural and functional similarities. Both cytokines are primarily produced by T-helper type 2 (Th2) cells, as well as by some innate immune cells, including basophils, mast cells, eosinophils, and macrophages. Both cytokines also share several biological functions. These include the promotion of Th2 cell differentiation, inducing the immunoglobulin switch from IgM to IgE in B cells, and recruiting and activating immune cells such as macrophages [9,10]. Consistent with these functional similarities, IL-4 and IL-13 exhibit approximately 30% amino acid sequence homology and share a common receptor, the type 2 IL-4 receptor [11]. Although IL-4 and IL-13 receptors share a common alpha chain (IL 4Rα), IL-4Rα also interacts with the gamma common (γc) chain to form the type 1 IL-4 receptor, while the type 2 IL-4/IL-13 receptor, IL-4Rα, binds to the IL-13Rα1 chain. Both receptor complexes can be activated by IL-4, resulting in JAK/STAT6-dependent signaling; this is also triggered by IL-13 binding but exclusively via the type 2 receptor complex. In contrast, IL-13 exhibits a higher binding affinity to the α2 chain of the IL-13 receptor (IL-13Rα2), which was previously specified as a decoy receptor for the internalization of excessive IL-13 [12,13]. Recently, a new signaling pathway for the IL-13Rα2 has been described in which activator protein 1 (AP1) acts as the signaling molecule to induce the production of TGF-β1 in macrophages and, therefore, mediates fibrosis [12]. It is widely accepted that, upon TGF-β1 stimulation, fibroblasts are activated and undergo a phenotypic transition into myofibroblasts accompanied by increased alpha-smooth muscle actin (α-SMA) production [14]. Recently, Marcelin et al. identified adipocyte progenitors (defined as PDGFRa+ and Cd9high) capable of adopting a myofibroblast-like phenotype leading to ECM deposition and fibrosis under a high-fed diet (HFD) [15]. Obesity and fibrotic WAT are also closely associated with increased pro-inflammatory ATMs, that primarily accumulate around dead adipocytes, forming crown-like structures (CLS) [16,17,18]. Here, most CLS-associated ATMs are lipid-associated macrophages (LAMs), a novel macrophage subset that are closely related to lipid metabolism and phagocytosis [19]. Interestingly, dying adipocytes induced the activation of macrophage-inducible C-type lectin (Mincle) exclusively in pro-inflammatory ATMs localized in CLS, thereby leading to myofibroblasts formation and ECM production [20]. 

On the other hand, fibrosis is closely linked with Th2 immune responses. The effects of IL-13 and IL-4 on fibrosis pathogenesis have been extensively studied, but their role in WAT fibrosis has not yet been elucidated. This study demonstrates, for the first time, that IL-13 and IL-4 stimulation results in a fibrotic phenotype in mouse WAT ex vivo and partly in vivo. Furthermore, we identify ATMs as the key driver mediating the IL 13/IL-4 signal. In contrast, data concerning human WAT cannot entirely support this conclusion. Indeed, visceral WAT expression revealed a positive correlation of IL-13/IL-4 receptors with fibrosis markers, but there was no positive correlation for IL-13 and IL-4, thereby suggesting a controversial role for IL-13 and IL-4 in human WAT fibrosis.

## 2. Results

### 2.1. IL-13 and IL-4 Induced WAT Fibrosis

**Figure 1:**
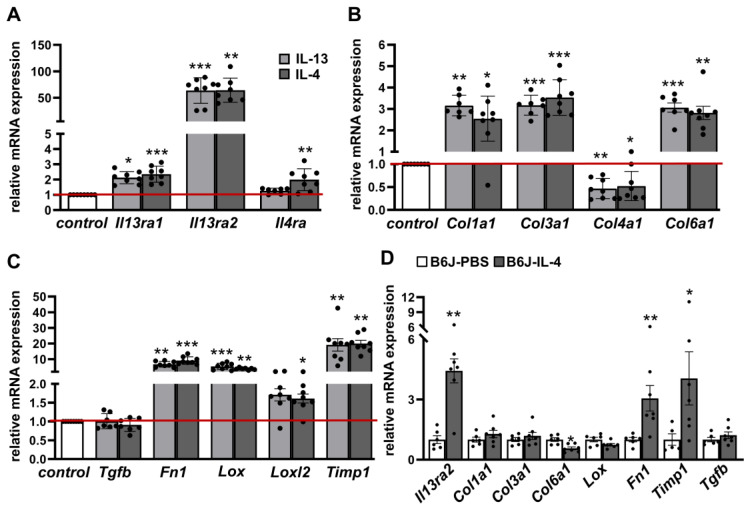
IL-13 and IL-4 induce fibrosis-related genes in adipose tissue of C3H mice and partial in WAT from B6J mice injected with IL-4. White adipose tissue (WAT) explants from C3H mice (n = 7–8) were stimulated either with IL-13 (50 ng/mL) or IL-4 (50 ng/mL) or non-stimulated (control) for four days. mRNA expression of cytokine receptors (**A**), collagens (**B**) and other fibrosis markers (**C**) are given as fold change compared to control condition. B6J mice were fed HFD over 6 weeks and were injected every two days with recombinant mouse IL-4 (66 μg/kg body weight) complexed with anti-IL-4 (333 μg/kg body weight) or PBS intraperitoneally (i.p.) for the final 2 weeks of feeding (n = 5–7). mRNA expression of Il13ra2 and fibrosis markers are given as fold change compared to PBS condition (**D**). Data represented as mean ± SEM. * *p*-value < 0.05; ** *p*-value < 0.01; *** *p*-value < 0.001. The red line indicates the level of the control group.

Several studies have demonstrated that IL-13 and IL-4 act as important inducers of fibrosis in several diseases. To study the impact of IL-13 and IL-4 on WAT fibrosis, we established an ex vivo culture system of WAT explants. Because varying susceptibility to WAT fibrosis has been reported in different mouse strains [21], we compared the commonly used fibrosis-prone C3H/HeOuj (C3H) mice with more fibrosis-resistant C57BL6/J (B6J) mice, as it has been shown that C3H mice possess more advanced WAT fibrosis after HFD feeding compared to B6J mice with similar body weight [15]. Studying the effect of cytokines solely on fibrosis development, we used WAT explants from lean chow-fed mice. After 4 days of IL-13 or IL-4 stimulation, the mRNA expression of their respective receptors *Il13ra1* and *Il13ra2* was significantly increased in both mouse strains, with C3H mice displaying higher upregulation in stimulated WAT explants (Figure 1A and Figure S1A). *Il4ra* expression was upregulated only after IL-4 treatment in C3H mice (Figure 1A). Interestingly, the IL-13-receptor *Il13ra2*, known as a decoy receptor, showed an upregulation up to 64-fold after stimulation with both cytokines, suggesting binding of excess cytokines, as previously described [13]. Importantly, IL-13 and IL-4 treatment upregulated the mRNA expression of many fibrosis-related and cross-linking enzyme genes (*Col1a1, Col3a1, Col6a1, Fn1, Lox, Loxl2, Timp1*) in both strains, whereby the expression levels were slightly higher in C3H mice (Figure 1B,C and Figure S1A). Counterintuitively, the expression level of *Tgfb1*, which is described to be upregulated in many fibrotic diseases [14], was not altered. Additionally, a concentration range from 1 to 250 ng/mL was used to test the dose dependency of IL-13 and IL-4 to induce fibrosis phenotype. We observed that only high concentrations (50 ng/mL and above) could induce expression of fibrosis-related genes (Figure S3A) and increase the amount of secreted fibronectin (Figure S3B). 

Next, we investigated our recent findings in an in vivo model. We used B6J mice receiving HFD for 6 weeks and recombinant mouse IL-4 complexed with IL-4 antibodies to stabilize IL-4 in vivo for the final two weeks of the feeding period. IL-4 treatment led to a significant increase of mRNA levels of the *Il13ra2* receptor (4.4-fold) as well as *Fn1* (3.1-fold) and *Timp1* (4.1-fold); expression levels other genes associated with fibrosis were unaltered (*Col1a1, Col3a1, Lox, Tgfb1*) or even decreased (*Col6a1*) (Figure 1D). This result partially reflects the findings from the ex vivo WAT explants.

**Figure 2:**
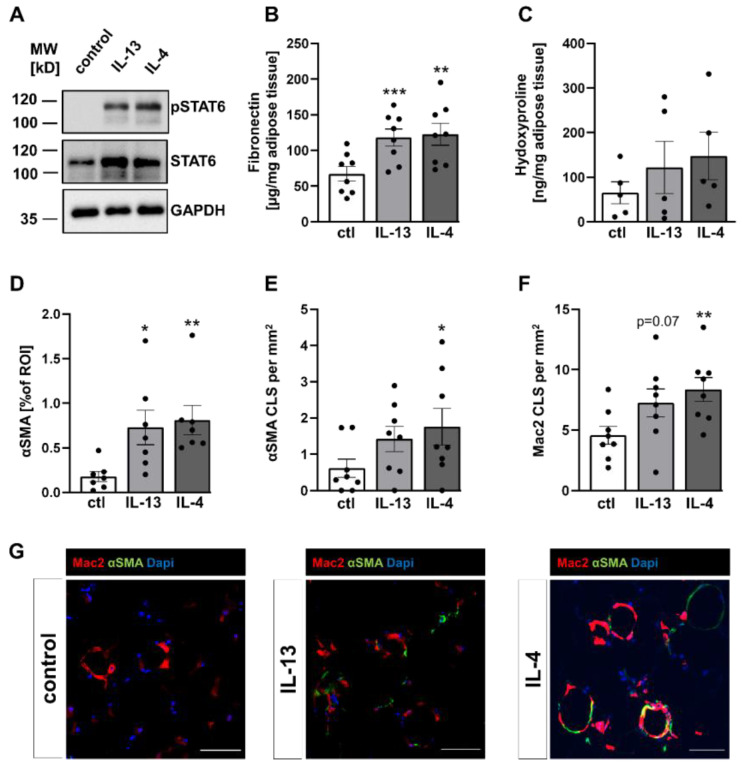
Increased STAT6 phosphorylation and fibrosis-associated proteins were induced by IL-13 and IL-4. WAT explants from C3H mice were stimulated with either IL-13 (50 ng/mL) or IL-4 (50 ng/mL) or non-stimulated (control) for four days. (**A**) A representative immunoblot from WAT explant lysates using antibodies against phosphorylated STAT6, total STAT6 and GAPDH to assess equal protein loading, from four independent experiments. (**B**) Levels of fibronectin were determined in supernatants of the explants (n = 8) by ELISA. (**C**) Collagen content in WAT explants was measured by hydroxyproline colorimetric assay (n = 5). (**D**) The area of α-SMA positive cells (n = 7), (**E**) the number of α-SMA positive crown-like structures (CLS, n = 8) and (**F**) the number of Mac-2 positive CLS (n = 8) in WAT explants were assessed by α-SMA (green) and Mac-2 (red) immunofluorescence staining. (**G**) Representative images of α-SMA and Mac-2 immunofluorescence staining. DAPI was used for nuclei counterstaining (blue). Data represented as mean ± SEM. * *p*-value < 0.05; ** *p*-value < 0.01; *** *p*-value < 0.001; scale bars = 50 µm.

Activation of type 1 and type 2 IL-4 receptors results in downstream phosphorylation of STAT6. To investigate STAT6-signaling under IL-13 and IL-4 stimulation in WAT explants, explants from C3H mice were stimulated in an ex vivo culture system for 4 days. We detected a distinct elevation of phosphorylated STAT6 in IL-13 and IL-4 treated AT explants when compared to control conditions (Figure 2A). In addition, total STAT6 levels were higher in the IL-13 and IL-4 treated AT explants. 

Referring to the increase of fibrosis-related genes, we wanted to ensure that changes in expression patterns were also reflected at the protein level. Therefore, we measured levels of fibronectin, one of the highest expressed genes after IL-13/IL-4 stimulation, in the supernatant of stimulated and non-stimulated explants. In agreement with expression data, the levels of secreted fibronectin in WAT explants of C3H mice were significantly increased by ~75% or 80% after IL-13 or IL-4 treatment, respectively (Figure 2B). Quantification of fibronectin levels in the supernatant of AT explants from B6J mice confirmed these data (Figure S1B). Furthermore, we also measured levels of the amino acid hydroxyproline, a broadly employed method used for quantifying collagen. In contrast to upregulated collagen expression, we only detected a non-significant increase of hydroxyproline in explants from C3H mice (Figure 2C and Figure S1C). 

In many tissues affected by fibrotic diseases, αSMA expression levels were used to identify myofibroblasts, the major collagen-producing cell type in fibrotic tissues [22,23]. Immunofluorescence analysis revealed that αSMA-positive cells were increased 3 to 4-fold after IL-13 or IL-4 stimulation in C3H mice (Figure 2D). In addition to single αSMA-positive cells, presumably myofibroblasts, we also observed an αSMA-positive circular staining pattern, resembling crown-like structures (CLS; an accumulation of ATMs surrounding dying adipocytes). The presence of ATMs within these circular structures was confirmed by the macrophage marker Mac-2. Hence, we defined these structures as αSMA-positive CLS (αSMA-CLS). We found significantly higher numbers of αSMA-CLS in AT explants stimulated with IL-4, whereas the increase of αSMA-CLS after IL-13 treatment did not reach significance (Figure 2E). Most interestingly, a few CLS-associated ATMs showed co-expression of αSMA (Figure 2G), while interstitial ATMs (outside of CLS) revealed no obvious αSMA expression, suggesting a correlation between ATMs in CLS and αSMA expression. In addition, the numbers of Mac-2 positive CLS were also elevated after IL-13 and IL-4 stimulation, raising the question of a positive correlation between number of CLS, as marker for inflammation, and αSMA-positive areas (Figure 2F and Figure S1D–F). In line with this, we observed a significant positive correlation for IL-4 induced Mac-2-CLS with αSMA-CLS in C3H mice (Figure S2). 

Overall, WAT explants stimulated with 50 ng/mL of IL-13 and IL-4 from C3H mice and B6J mice are characterized by an increased propensity for WAT fibrosis, whereas WAT fibrosis is more pronounced in C3H animals. Furthermore, in vivo data from B6J mice injected with IL-4 partially confirmed its effects on fibrosis marker expression. 

### 2.2. IL-4Rα-Chain Is Required for IL-13 and IL-4 Induced Fibrosis

**Figure 3:**
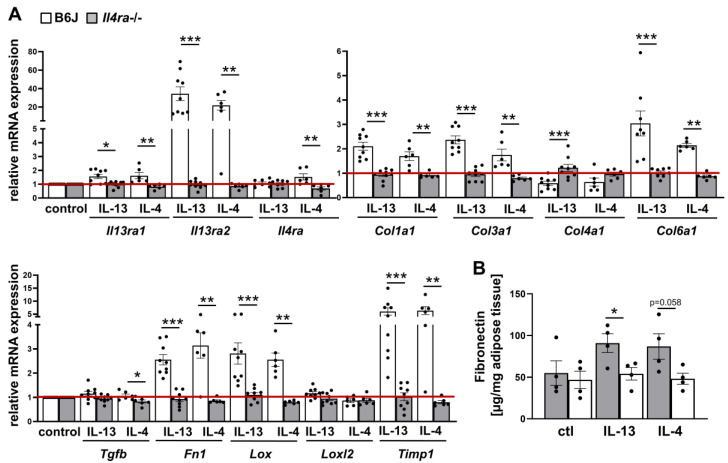
WAT explants from *Il4ra−/−* mice show no response to IL-13 and IL-4 stimulation. WAT explants from *Il4ra−/−* mice (n = 6–9) and control B6J mice (n = 6–9) were stimulated with either IL-13 (50 ng/mL) or IL-4 (50 ng/mL) or not (control) for four days. (**A**) mRNA expression of cytokine receptors and fibrosis-related genes were determined in stimulated explants and represented as relative changes to the control group (B6J). (**B**) Fibronectin content was measured in the supernatant of cultured explants by ELISA. All data were represented as mean ± SEM. * *p*-value < 0.05; ** *p*-value < 0.01; *** *p*-value < 0.001. The red line indicates the level of the control group.

Given the significant induction of fibrosis in response to IL-13 and IL-4, we next investigated which receptor was mediating this effect in WAT. There are three potential receptors: the type 1 and type 2 IL-4 receptor, which share the common receptor IL-4Rα-chain and can be activated by IL-4 (type 1, type 2) or IL-13 (type 2), and IL-13Rα2, which is activated by IL-13 only [24]. Since both cytokines can induce fibrosis, we hypothesized that the IL4Rα-chain could be the major receptor chain for IL-13 and IL-4 signaling. Hence, we used WAT explants from mice lacking the IL4rα-chain (*Il4ra−/−)* and age-matched B6J control mice. WAT explants were stimulated in an ex vivo model with IL-13 and IL-4 for 4 days as described above. In B6J control mice, we determined similar levels of mRNA expression for receptor genes (*Il13ra1, Il13ra2, Il4ra*) and fibrosis as well as cross-linking enzyme genes (*Col1a1, Col3a1, Col6a1, Fn1, Lox, Timp1*) (Figure 3A) as observed in B6J mice described in Figure S1A.

However, WAT explants from *Il4ra−/−* mice showed no response to IL-13 and IL-4 when compared to explants from age-matched wild-type B6J mice (Figure 3A). Moreover, the abundance of secreted fibronectin was quantified. In line with mRNA expression, the level of fibronectin did not increase upon IL-13 and IL-4 treatment in *Il4ra* deficient WAT explants (Figure 3B); this confirms that the IL4rα-chain is absolutely required for IL-13 and IL-4 induced fibrosis.

### 2.3. IL-13 and IL-4 Induced Fibrosis Phenotype Depends on ATMs

Next, we set out to examine the mediator cells of IL-13 and IL-4 induced fibrosis in WAT. We characterized cultured adipocytes and macrophages by analyzing their gene expression profile. Differentiated 3T3-L1 adipocytes were stimulated with 50 ng/mL IL--13 or IL-4 over 48 h, respectively and with 5 ng/mL TGF-β1 as a positive control. Analysis of mRNA expression revealed no induction of fibrosis-related genes under IL-13 and IL-4 stimulation. TGF-β1, as a well-known stimulus for fibrosis, increased mRNA expression of some genes, including *Col1a1, Fn1, Lox*, and *Timp1* (Figure S4A). 

**Figure 4:**
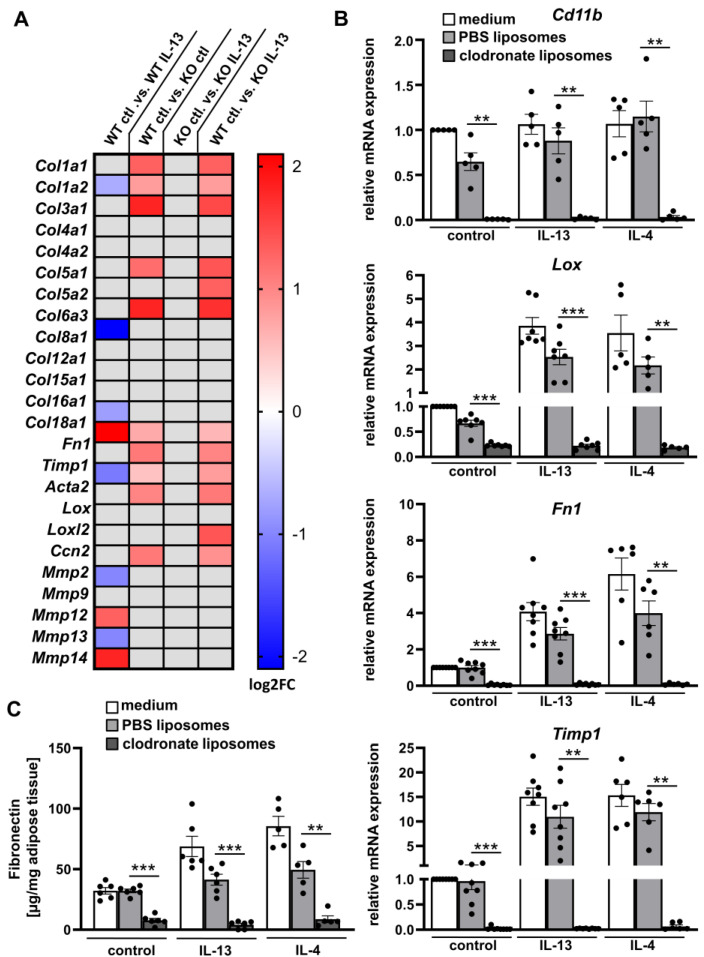
Adipose tissue fibrosis is dependent on adipose tissue macrophages (ATMs). (**A**) BMDMs were isolated from *Il4ra−/−* mice (KO, n = 4) and *Il4ra+/+* littermate controls (WT, n = 4) and stimulated with IL-13 (20 ng/mL) for 48 h. Differential gene expressions (DEGs) were determined from RNA bulk sequencing data and Log2FC of fibrosis-related genes were represented in a heat map; gray boxes illustrate non-significant data (FDR > 0.05) (**B**) Explants from B6J mice (n = 5–8) treated with clodronate or PBS liposomes or non-treated (medium) were stimulated for four days with 50 ng/mL IL-13 or IL-4, respectively. mRNA expression of the macrophage marker (*Cd11b*) and fibrosis markers are shown as fold change compared to control conditions (medium, non-stimulated). (**C**) Levels of fibronectin were determined in supernatants of WAT explants (n = 6) by ELISA. All data were represented as mean ± SEM. ** *p*-value < 0.01; *** *p*-value < 0.001.

To examine the impact of macrophages, we stimulated bone marrow derived macrophages (BMDMs) from *Il4ra*−/− deficient mice and littermate wild-type (WT) control mice with IL-13 (20 ng/mL) over 48 h and analyzed them using RNA sequencing (Figure 4A). Interestingly, we only observed an increased mRNA expression of *Fn1* in response to IL-13 in WT BMDMs; other fibrosis-related genes were not altered. In *Il4ra*−/− deficient BMDMs, fibrosis-related genes were not upregulated in response to IL-13. In contrast, comparing only BMDMs from WT and *Il4ra*−/− deficient mice without stimulation, we determined an increase in some fibrosis-related genes such as *Col1a1, Col1a2, Col3a1, Col5a1, Col6a3, Fn1, Timp1, Acta2* and *Lox* in *Il4ra*−/− deficient BMDMs, suggesting a basal difference between WT und *Il4ra*−/− deficient BMDMs; this was not altered even after IL-13 stimulation. Hence, basal gene expression of fibrosis genes in cultured macrophages depend on *Il4r* expression. However, to mimic fibrosis induction due to IL-13 stimulation, more complex culture systems (such as WAT explants) are needed, presumably due to the lack of myofibroblasts or adipocyte progenitors as additional mediators. 

Therefore, we further examined the role of ATMs in IL-4/IL-13-induced WAT fibrosis using WAT explants. A suitable mechanism to eliminate macrophages in vivo is treatment with clodronate liposomes [25]. First, we confirmed ATMs depletion by mRNA expression of the macrophage marker Cd11b (*Itgam*). *Cd11b* expression was only slightly decreased in WAT explants treated with PBS liposomes or IL-13 and unaltered after IL-4 stimulation, while clodronate liposomes dramatically reduced the expression of *Cd11b* in all conditions (Figure 4B). This finding demonstrates that clodronate liposomes can efficiently deplete ATMs in our ex vivo model. We further characterized ATMs depleted WAT explants regarding the expression of fibrosis-related genes upon IL-13 or IL-4 treatment. Interestingly, *Lox, Fn1*, and *Timp1* mRNA levels were decreased by > 90% when compared to PBS control liposomes (Figure 4B). In line with the expression data, secreted fibronectin abundance was also reduced by ~90% and 80% in IL-13 or IL-4 stimulated WAT explants treated with clodronate liposomes (Figure 4C), respectively. These data clearly support the important role of ATMs in WAT fibrosis.

### 2.4. IL-13 and IL-4 Do Not Positively Correlate with Fibrosis Markers and Parameters Associated with Obesity in Human WAT

Finally, we performed a gene correlation analysis of *IL13* and *IL4* expression with cytokine receptors, fibrosis markers, IL-13/IL-4 signaling components, and metabolic parameters in human WAT to address the clinical relevance of our data and potential association with parameters of obesity. We analyzed the mRNA expression of visceral and subcutaneous WAT samples from 1553 adult patients from the Leipzig Obesity BioBank and adjusted for sex, BMI, and age. Most of the patients in this cohort were obese (BMI ≥ 30, N = 1470), which increases the occurrence of WAT fibrosis. 

**Figure 5:**
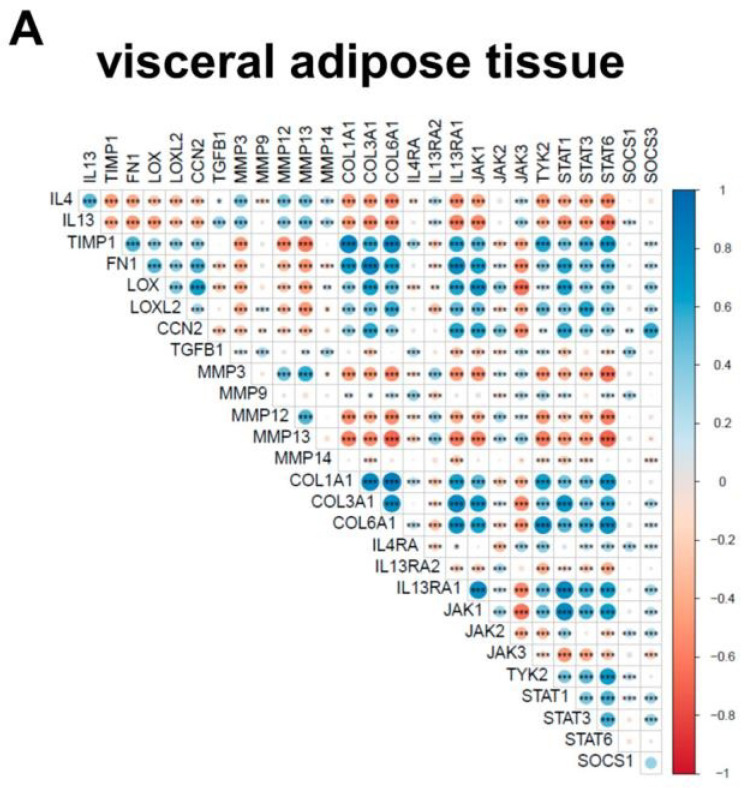

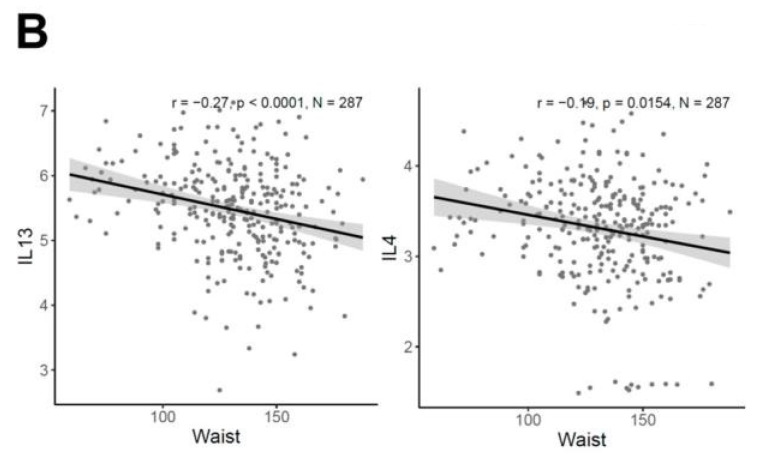
Human gene correlation analysis of visceral adipose tissues. The presented data are RNA-Seq data from human visceral adipose tissue samples (n = 1553). (**A**) Correlation analysis of fibrotic-related genes as well as *IL13* and *IL4* in visceral WAT. (**B**) Significant negative correlation of IL13 and IL4 with waist circumference (N = 287). Positive correlations are shown in blue while negative correlations are represented in red. The size of the dot refers to the degree of correlation; * *p*-value < 0.05; ** *p*-value < 0.01; *** *p*-value < 0.001.

*IL13* and *IL4* expression positively correlated with *IL13RA2*, *JAK3*, *TGFB1* and several matrix metalloproteinases (MMP) in visceral and subcutaneous WAT (Figure 5A and Figure S5). In contrast, other fibrosis markers (*TIMP1*, *FN1*, *LOX*, *LOXL2*, *CCN2*, *COL1A1*, *COL3A1,* and *COL6A1*), showed an inverse correlation in both WAT tissues with *IL4* and *IL13* expression. However, *IL13RA1* revealed a positive correlation to all fibrosis markers (except *TGFB1*) and *IL4RA* only for a few (*TIMP1*, *TGFB1*, *COL1A1*, and *COL6A1*) in visceral WAT, consistent with the results in mouse WAT explants. We further correlated *IL13* and *IL4* expression with metabolic parameters in visceral and subcutaneous WAT. For *IL13*, we found a significant inverse correlation to body weight, body fat, and waist circumference, whereas *IL4* expression inversely correlated with waist circumference, fasting plasma glucose (FPI), and homeostatic model assessment for insulin resistance (HOMA-IR) in visceral WAT (Figure 5B, Table S1). We observed no significant correlations for *IL13* and *IL4* in subcutaneous WAT (Table S2). It is worth nothing that several fibrosis markers, including *TIMP1*, *FN1*, *LOX*, *CCN1*, *COL1A1*, *COL3A1*, and *COL6A1*, positively correlated with either body fat or/and waist circumference (Tables S1 and S2). 

Overall, the correlation data from a large human WAT Obesity BioBank offered conflicting results, as discussed below.

## 3. Discussion

Numerous studies have focused on WAT fibrosis, a consequence of a chronic inflamed and progressive metabolic imbalance during obesity. WAT fibrosis is characterized by excessive accumulation of ECM to counterbalance the arising WAT dysfunction [18,26]. However, the underlying cellular and molecular mechanisms are still ill-defined. 

In this study, we aimed to clarify whether IL-13 and/or IL-4, well-known fibrosis inducers in various tissues, can also promote fibrosis in WAT. Therefore, we used an organotypic culture model that preserves the physiological function of WAT and the in vivo crosstalk between various types of cells [27]. Expression of several fibrosis markers, as well as levels of fibronectin and αSMA protein levels, were increased upon IL-13 and IL-4 stimulation, but this phenotype was entirely absent in WAT lacking *Il4ra*. This suggests that IL-13 and IL-4 are also involved in fibrotic remodeling in WAT depending on the IL-4 receptor α-chain. Notably, only concentrations above 50 ng/mL could induce the fibrotic phenotype, which does not reflect physiological cytokine concentrations [28,29]. An in vivo mouse model with repeated IL-4 injections partly confirmed our results from the ex vivo WAT explants. Here, we only observed an increase of two fibrosis markers (*Timp1* and *Fn1*), while other key markers, such as collagens, were not altered, suggesting that the in vivo effect of IL-4 on WAT fibrosis is not very pronounced or only occurs after chronic stimulation over longer time periods. Even though the injected IL-4 concentration was very high (66 µg/kg body weight), the expression levels of *Timp1* and *Fn1* were not comparable with those of ex vivo results. There are very few studies regarding fibrosis in IL-4- or IL-13-injected mice but, in contrast to our results, Kaviratne et al. provided evidence that intraperitoneal injections of IL-13 can lead to liver fibrosis by upregulating hepatic *Col1*, *Col3*, and *Timp1* expression [30]. However, we assume that physiological concentrations of IL-13 and IL-4 may not induce WAT fibrosis within a short time frame, but maybe over longer periods. Interestingly, we found that *Il13ra2*, an exclusive IL-13 decoy receptor, was induced even upon IL-4 stimulation, both ex vivo and in vivo. We hypothesize that a negative feedback mechanism may exist, by which IL-4 reduces an overactive Th2 response via the expression of *Il13ra2*. This feedback mechanism appears to be regulated by the α-chain of the IL-4 receptor, as *Il4ra* deficient mice do not show an increase of *Il13ra2* upon IL-4/IL-13 stimulation.

Th2 inflammation is considered to be one of the primary mediators in the pathogenesis of several fibrotic disorders. Surprisingly, the mechanism by which Th2 cytokines mediate fibrosis has not been consistently addressed. Several studies have found that IL-13 and IL-4 can activate fibroblasts and stimulate collagen deposition, as has already been shown for the prominent fibrosis inducer TGF-β1 [29,31,32]. Depending on tissues and cell types, fibrogenic effects of IL-13 and IL-4 are mediated by TGF-β1 induction [32,33] or conveyed independently [30], suggesting a broad spectrum of Th2-effective pathways. Previously published articles have already demonstrated that macrophages stimulated with IL-4 and/or IL-13 can produce different ECM components, affecting the biomechanical properties of a tissue; this is clearly seen in fibrotic diseases [34,35,36,37,38]. Here, we showed that WAT explants stimulated with IL-13 or IL-4 increased the expression of fibrosis genes as well as fibronectin secretion. Importantly, *Tgfb1* expression was not altered, indicating a TGF-β1-independent activation of WAT fibrosis through IL-13 and IL-4. However, the peak of *Tgfb1* induction might be earlier than our observation point at 48 h. As shown in macrophages and fibroblasts, IL-13 and IL-4 are also potent inducers of arginase expression, which converts L-arginine into L-ornithine and urea. Of note, L-ornithine is a precursor of proline, which promotes collagen synthesis and cell proliferation [39,40]. While we demonstrated an increase of collagen expression upon IL-13 and IL-4 stimulation in WAT explants, we could also observe an increased number of CLS containing Mac-2-positive ATMs. This observation is in line with our previous finding that M2-like ATMs possess the ability to proliferate within CLS [41]. Moreover, the αSMA positive area that increased upon IL-13 or IL-4 stimulation also appears to be localized in CLS; this could be evidence for cellular crosstalk within CLS. The recently found Mincle expression, localized only in pro-inflammatory ATMs within CLS, supports this assumption. As endogenous ligands (e.g., free fatty acids), released from dying adipocytes, Mincle is activated in ATMs, which in turn leads to expression of fibrosis-related genes and myofibroblasts activation [20,42]. Moreover, Mincle KO mice are protected from obesity-induced CLS formation and WAT fibrosis, indicating that Mincle plays a role in the crosstalk between adipocytes and macrophages within CLS [20]. A newly found subset of macrophages, called LAMs, were also localized within CLS, thereby expressing high levels of Cd9 and Trem2. Of note, TREM2 deficiency exacerbates WAT hypertrophy and insulin resistance by preventing LAM formation in response to a HFD [19]. This suggests an important role for LAMs in attenuating metabolic remodeling in obese WAT. It is worth noting that this subpopulation is also classified as pro-fibrotic, as TREM2^+^CD9^+^ macrophages expand in liver fibrosis [43]. Further investigations are needed to clarify if IL-13 or IL-4 have an impact on the fibrotic effects associated with LAMs within CLS.

Interestingly, Itoh et al. found that αSMA-positive myofibroblasts and collagen depositions are localized in proximity to hepatic CLS in a NASH mouse model [44]. This raises the question of the origin of αSMA-positive myofibroblasts within CLS. The origin of myofibroblasts in fibrotic WAT has been the subject of intensive investigation but remains controversial. For a long time, tissue-resident fibroblasts were thought to be the main precursor of myofibroblasts in WAT. However, recent studies have implicated an unexpected role of mature adipocytes, which can acquire a myofibroblast phenotype under fibrotic stimuli [45,46]. This process has been described as adipocyte mesenchymal transition [47]. In addition, Marcelin et al. have shown a phenotypical switch of adipose progenitors, classified as Cd9highPDGFRα+ progenitors, to myofibroblasts, which promote ECM deposition and WAT fibrosis [15]. However, we detected αSMA-positive myofibroblasts within CLS formed by ATMs as well as CLS formed only by myofibroblasts. It should be mentioned that only IL-4 stimulation significantly increased the number of αSMA-positive CLS, whereas the increase with IL-13 stimulation did not reach significance. This lower efficacy of IL-13 may be due to the higher binding affinity of IL-13 to IL-13Rα2 compared with IL-13Rα1, which suppresses the impact of IL-13 at the IL-4 receptor type II. Regardless, the cellular origin of IL-13 and IL-4 induced myofibroblasts remains unclear to date and warrants further investigation. There are multiple theories regarding the phenotypical switch of adipocytes within fibrosis progression. One theory states that adipocytes dedifferentiate into mesenchymal progenitor cells upon systemic metabolic stress or hypoxia and then differentiate into myofibroblasts under fibrotic stimuli such as TGF-β1 [48]. On the other hand, these conditions could also cause ATM localization around dying adipocytes and thereby initiate the differentiation of adipose progenitors into myofibroblasts [47]. However, we studied the impact of ATMs on IL-13 and IL-4 induced fibrosis. Interestingly, the treatment with clodronate liposomes decreased the expression of fibrotic markers up to 99% and fibronectin abundance up to 90%. Thus, we postulate a direct crosstalk of ATMs to other cells, promoting the phenotypical switch towards myofibroblasts. Our finding that isolated macrophages and mature adipocytes do not respond upon IL-13 and IL-4 stimulation emphasizes this assumption. It is worth mentioning that mature adipocytes reduce *Il4ra* expression during the early phase of differentiation [49]; thus, using preadipocytes would be more physiological. In line with this, primary human adipocytes and preadipocytes co-cultured with THP-1 macrophages strongly increased collagen VI expression, most notably through M2-polarized macrophages [50]. Direct effects on the differentiation of SGBS preadipocytes by macrophages were reported by Sarsenbayeva et al., whereby the presence of macrophages induced αSMA expression in SGBS preadipocytes [51]. 

Interestingly, IL-4 can inhibit adipogenesis at the early phase of adipocyte differentiation through the STAT6 pathway [49]. This could be a possible mechanism by which IL-4 (and IL-13) increases the number of preadipocytes and, thereafter, myofibroblasts. 

In human subjects, there are numerous studies indicating the significance of IL-13 and IL-4 for fibrotic diseases in different tissues. A study on 611 patients with *Schistosoma japonicum* infection confirmed the association of Th2 cytokines, including IL-4 and IL-13, with liver fibrosis [52]. Moreover, lung biopsies from patients suffering from idiopathic pulmonary fibrosis also exhibited elevated levels of IL-13 and IL-4 [53]. In this study, we investigated visceral and subcutaneous WAT expression from 1553 patients with a wide range of metabolic data. Regarding the expression of *IL13* and *IL4*, we found no evidence of a positive correlation with any fibrosis markers or metabolic parameters suggesting a controversial role of IL-13 and IL-4 in human WAT fibrosis. Although most of the patients were obese (N = 1470), indicating a higher risk for WAT fibrosis, the histological evidence for confirmed fibrosis was not available and needs to be provided in future studies. Of note, Kwon et al. found an increase of *IL13* expression in WAT of obese patients and HFD-fed mice compared to lean conditions; this was mediated by adipocytes, presumably to counterbalance tissue inflammation [54]. Moreover, IL-4 was also elevated in obese patients [55], suggesting the participation of IL-4 in the process of diet-induced obesity and metabolism. Controversially, we observed an inverse correlation for *IL13* with body weight, body fat, and waist circumference. In line with this finding, *IL4* correlated inversely with FPI and HOMA-IR as well as waist circumference. Supporting this data, Chang et al. reported an improved glucose tolerance and insulin sensitivity in mice injected with recombinant IL-4 during HFD feeding [56]. In contrast to our finding of the inverse correlations of *IL13* and *IL4*, we observed positive correlations of several collagens and other fibrotic marker genes with receptors of IL-13 and IL-4 as well as body fat and waist circumference in visceral and partly subcutaneous WAT. This result is in line with previous findings, showing that *COL6a3* expression increases with obesity and correlates positively with visceral fat mass and BMI [57,58].

While our analyses seem to suggest a controversial role for *IL13* and *IL4*, we acknowledge that no firm conclusions can be drawn from these correlations regarding the functional significance of IL-13 and IL-4 in WAT fibrosis. Therefore, further investigations are required to clarify pathophysiological mechanisms behind WAT fibrosis and concomitantly the role of IL-13 and IL-4 in this context.

## 4. Materials and Methods

### 4.1. Animals

All mice were maintained in temperature-controlled, pathogen-free facilities with a 12 h light/dark cycle and given free access to food and water. Male C57BL/6J mice (named B6J), Il4ra−/− mice, and Il4ra+/+ littermate controls were housed at the Universities of Halle and Leipzig. Fibrosis sensitive mouse strain C3H/HeOuJ (hereafter named C3H) were purchased from Jackson Laboratories (2498063-66). Male animals were fed a normal chow diet (9% kcal fat, Ssniff Spezialdiäten; Germany) and euthanized at the age of 18 to 22 weeks. Male C57BL/6J mice were fed an HFD (60% kcal deriving from fat, Research Diets, Inc.) over 6 weeks at the University of Dresden. Mice were injected with recombinant mouse IL-4 (66 μg/kg body weight) complexed with anti-IL-4 (333 μg/kg body weight) or PBS i.p. every other day for the final two weeks of the HFD feeding. Experiments were performed in accordance with the rules of animal care issued by the local government authorities and were approved by the animal care committee of the Universities of Halle, Leipzig, and Dresden, as well as by the state of Saxony and Saxony-Anhalt (Bezirksregierung Leipzig, Bezirksregierung Halle, Germany, T11/21, I11M25, Landesdirektion Sachsen, Germany, TVV57/2018).

### 4.2. Adipose Tissue Explant Culture

Epididymal WAT of male mice was used to generate ex vivo WAT organotypic cultures (WAT explants) [59]. Therefore, under sterile conditions, the dissected WAT was cut into small pieces (<1 mm³) at 37 °C in PBS. 30–50 mg of these explants were transferred to six-well plates, overcasted with cell culture inserts (Merck Millipore, Darmstadt, Germany) and cultured in RPMI cell culture medium supplemented with 10% fetal bovine serum (FBS), 1% insulin-transferrin-selenium mixture, and 1% penicillin/streptomycin (all from Thermo Fisher Scientific, Waltham, MA, USA) for 7 days at 37 °C with 5% CO_2_. After 3 days, AT explants were stimulated with 50 ng/mL of IL-13 or IL-4. On day 7, WAT explants were snap frozen in liquid nitrogen for RNA isolation or hydroxyproline assays and the supernatant was collected to quantify the volume. Testing the effect of different cytokine concentrations, WAT explants were stimulated in a range from 1 ng/mL up to 250 ng/mL of IL-13 or IL-4. 

### 4.3. Clodronate Liposomes Treatment

A suitable mechanism to deplete macrophages in vivo is treatment with clodronate liposomes [25]. A high intracellular concentration of clodronate initiates programmed cell death and subsequently leads to the elimination of the macrophages [60]. Free clodronate does not easily cross cell membranes, so we used lipid vesicles encapsulating clodronate or an aqueous PBS solution (control) which were ingested by macrophages (Liposoma, The Netherlands). For macrophage depletion in AT explants, we used RPMI cell culture medium supplemented with 10% FBS, 1% insulin-transferrin-selenium mixture, 1% penicillin/streptomycin with 1 mg/mL clodronate, or PBS liposomes. First, the explants were transferred to tubes containing clodronate or PBS liposomes media followed by a rotation time at 37 °C for 60 min. Then, explants were cultivated as described above. 

### 4.4. Culture of Bone Marrow-Derived Macrophages (BMDMs)

BMDMs were generated from bone marrow cells of *Il4ra−/−* mice and littermate controls (*Il4ra+/+*). Bone marrow cells were collected by flushing tibias and femurs with PBS and centrifuged for 10 min at 300 g [61]. Subsequently, the cells were differentiated into macrophages in BMDM medium (RPMI1640 medium supplemented with 10% FBS, 1 mM GlutaMax, 1% penicillin/streptomycin, all from Thermo Fisher Scientific, Waltham, MA, USA) and 20 ng/mL M-CSF (PeproTech, Hamburg, Germany) at 37 °C with 5% CO_2_. After 7 days, BMDMs were harvested with an ice-cold 1 mM EDTA/PBS solution and 5 × 10^5^ cell/mL were seeded in 1 mL BMDM medium. For cytokine stimulation, BMDMs were treated 24 h after seeding with 20 ng/mL IL-13 (PeproTech, Hamburg, Germany) for 48 h. 

### 4.5. Culture of 3T3-L1 Cells

Murine 3T3-L1 cells were cultivated in DMEM high glucose supplement with 10% FBS and 1% penicillin/streptomycin (all from Thermo Fisher Scientific, Waltham, MA, USA) at 37 °C and 5% CO2 [62]. After 5 days, 3T3-L1 cells were seeded into 6-well plates at a density of 1 × 105 cells per well. 3T3-L1 cells were differentiated into adipocytes by treating confluent cells with DMEM, 10% FBS, 1% penicillin/streptomycin, 0.5 mM 3-isobutyl-1-methylxanthine (IBMX), 0.25 μM Dexamethasone, 0.2 µM Insulin, and 2 μM Rosiglitazone. On day 3, the media was switched to DMEM, 10% FBS, 1% penicillin/streptomycin, and 0.2 µM Insulin. From day 6 on, cells were further cultured under standard conditions. Cytokine stimulation on day 9 was performed with 50 ng/mL IL-13 or IL-4 or 5 ng/mL Tgf-β1 (PeproTech, Hamburg, Germany) for 48 h.

### 4.6. RNA Isolation and Quantitative RT-PCR

Total RNA was extracted from epididymal WAT and AT explants using the RNeasy Lipid Tissue Mini Kit (Qiagen, Hilden, Germany) followed by cDNA synthesis from 1 µg of total RNA with the First Strand cDNA Synthetis Kit (New England Biolabs) [61]. Quantitative real-time PCR was performed by using a SYBR green qPCR Master Mix (Thermo) and a ViiA™ 7 Real-Time PCR System (Thermo Fisher Scientific, Waltham, MA, USA). Primer sequences are listed in Table S3 in the supplementary materials. Gene expression levels were calculated through the ∆Ct method by using Ipo8 as a housekeeping gene. For a better visualization, the values of the control group were set to 1 and mRNA expression was given as fold change compared to the control group. 

### 4.7. Western Blotting

Western blotting was performed as recently described [51]. Total protein from WAT explants was extracted using an extraction buffer (Thermo Fisher Scientific, Waltham, MA, USA) supplemented with a protease and phosphatase inhibitor cocktail (Roche, Basel, Switzerland). Protein concentration was measured with the DC protein assay (Bio-Rad, Hercules, USA) according to the manufacturer’s instructions. Equal amounts of protein (25 μg) were loaded to SDS-PAGE and transferred to a PVDF membrane. After blocking with 5% bovine serum albumin (BSA) in TBS/T, blots were incubated with primary antibodies against pSTAT6 (1:1000, #56554; Cell Signaling Technology, Boston, MA, USA), STAT6 (1:1000, #5397; Cell Signaling Technology, Boston, MA, USA), and GAPDH (1:1000, #3686; Cell Signaling Technology, Boston, MA, USA) for 16 h. After an incubation with a HRP-conjugated secondary antibody, immunoreactions were detected by visualizing the peroxidase activity with an ECL Kit (Pierce™ ECL Western Blotting Substrate, Thermo Fisher Scientific, Waltham, MA, USA). For reloading the membrane with primary antibody, blots were stripped with western blot stripping buffer (Thermo Fisher Scientific, Waltham, MA, USA) according to the manufacturer’s instruction.

### 4.8. Collagen Content

Hydroxyproline measurement was performed using a hydroxyproline colorimetric assay (BioVision, Waltham, MA, USA) [15]. Snap frozen WAT explants were weighted and homogenized in water (100 mL water/10 mg WAT explants) using tissue homogenization (Precellys 24 Tissue Homogenizer, Bertin technologies, Montigny-le-Bretonneux, France). Homogenates (100 µL) were heated with 100 µL 12 M HCl for 3 h at 120 °C; then, 10 µL of the supernatant and a hydroxyproline standard were evaporated before being incubated with chloramine-T and p-dimethyl amino-benzaldehyde (DMAB) reagent at 60 °C for 90 min. The absorbance was read at 560 nm and the concentration was calculated using the standard curve and weights from WAT explants. 

### 4.9. Fibronectin Content

The measurement of fibronectin levels was performed using a specific fibronectin ELISA kit (Fibronectin mouse ELISA kit 108849, Abcam, Cambridge, UK) [63]. Cell culture media from WAT explants were centrifuged to remove debris, and supernatants were collected and diluted 1:50 in the diluent buffer provided with the kit. The fibronectin ELISA was performed according to the manufacturer’s protocol. 

### 4.10. Immunofluorescence

For immunofluorescence staining, AT explants were fixed in zinc formalin for 2 h and embedded in paraffin [64]. Paraffin sections were deparaffinized, unmasked in a pressure cooker at 120 °C in Tris/EDTA buffer (pH 9.5), and washed in PBS with 0.3% Triton. Unspecific binding sites were blocked using 1% BSA in PBS with 0.3% Triton for 1 h at room temperature. Sections were incubated overnight at 4 °C with primary antibodies against the macrophage marker Mac-2 (1:1000; Cedarlane CL8942AP, Canada) and the myofibroblast marker αSMA (1:200; Cell Signaling 19245, USA), followed by appropriate fluorochrome-conjugated secondary antibodies (1:200; Invitrogen; Waltham, MA, USA) and Hoechst for nuclear staining (1:10,000 in PBS, Life Technologies, Carlsbad, CA, USA). Control stainings were performed following the same routines without primary antibodies. Images were taken using an Olympus BX40 epifluorescence microscope. For αSMA quantification, a region of interest (ROI) was defined around the intact WAT explant and the αSMA positive area, above a determined threshold, within this ROI was then measured using ImageJ software 1.53 k. The threshold was determined in ROI sections of the control staining to exclude unspecific background noise. αSMA and Mac-2 positive CLS were quantified by counting in 10 randomly chosen fields in one section per animal, whereby CLS were defined as adipocytes surrounded by Mac-2 or αSMA positive cells. 

### 4.11. RNA Bulk Sequencing from BMDMs and Differential Gene Expression Analysis

BMDMs were snap frozen in TRIzol (Quiagen, Hilden, Germany) and RNA bulk sequencing was performed by Single Cell Discoveries (Utrecht, The Netherlands). RNA extraction and library preparation followed the CELseq2 protocol [65] with a sequencing depth of 10 million reads/sample. For RNA-sequencing data analyses, low quality read ends were clipped off using Cutadapt (v 1.14) [66]. Subsequently, the processed sequencing reads were aligned to the murine reference genome (UCSC mm39) using HiSat2 (v 2.1.0) [67]. Samtools (v 1.10) was used to extract primary alignments and to index the resulting bam-files [68]. FeatureCounts (v 2.0.0) was used for summarizing gene-mapped reads [69]. ENSEMBL (GRCm39 v105) was used as annotation basis [70]. Differential gene expression (DGE) was determined using the R package edgeR (v 3.38.4) utilizing trimmed mean of M-values (TMM) normalization [71,72]. In order to account for biases in the expression values introduced by different batches, blocking was used to reduce these effects. A false discovery rate (FDR) value below 0.05 was considered as threshold for the determination of differential gene expression.

### 4.12. RNA Bulk Sequencing from Human Data

The human cross-sectional cohort from the Leipzig Obesity BioBank comprises 1553 individuals. Omental visceral and abdominal subcutaneous WAT samples were collected from each individual; these individuals were either non-obese (N = 83; 50.6% female; age: 64.2 ± 13.4 years old; BMI: 25.4 ± 2.7 kg/m²) or obese (N = 1470; 70.8% female; age: 47.2 ± 11.9 years old; BMI: 48.9 ± 8.5 kg/m^2^). Tissue samples were collected during elective laparoscopic abdominal surgery, as previously described [73]. Measurements of metabolic parameters and body composition were performed as described in detail before [74]. Bulk RNA-seq data were conducted with a SMARTseq protocol [75]. All libraries were sequenced on a Novaseq 6000 instrument at the Functional Genomics Center Zurich (FGCZ). Adapter and quality trimming of the raw reads were performed using fastp v0.20.0 [76] (minimum read length of 18 nts, quality cut-off of 20). Sequence pseudo alignment of the resulting high-quality reads to the human reference genome (build GRCh38.p13) and gene level expression quantification (gene model definition from GENCODE release 32) was computed using Kallisto v0.46 [77]. Samples with more than 20 million mapped read counts were downsampled to 20 million read counts using the subsampleCountMatrix function of the R package ezRun v3.14.1 (https://github.com/uzh/ezRun, accessed on 23 March 2022). Count data were homoscedastic normalized with respect to library size using the variance stabilizing transformation from DESeq2 v1.32.084 [78] and adjusted for age, BMI, and sex.

### 4.13. Statistical Analysis

Statistical analyses were performed using GraphPad Prism9 (GraphPad Software, Inc., New York, NY, USA). At first, data were checked for statistical outliers using the ROUT test (Q = 1%). Normality and homogeneity of variance were assessed using the Shapiro–Wilk test and F-test, respectively. For two-group analyses with normally distributed data, an unpaired Student’s *t*-test (data with equal variances) or Welch *t*-test (data with unequal variances) were used. For non-normally distributed data, a Mann–Whitney U test was used for two-group analyses. To compare more than two groups, a one-way ANOVA followed by Dunnett’s multiple comparison test was assessed for normally distributed data. For non-normally distributed data of more than two groups, a Kruskal–Wallis test followed by Dunn’s multiple comparison test was performed. Fibronectin and collagen content was analyzed by pairing one-way ANOVA with (data with unequal variances) or without (data with equal variances) followed by Geisser–Greenhouse correction or Dunnett’s multiple comparison test. A Friedman test was used for non-normally distributed and repeated-measured data followed by Dunn’s multiple comparison test. The data are expressed as means ± SEM and sample size is given in the respective figure legends. A Pearson correlation analysis was performed between the number of αSMA-positive CLS and Mac2-positive CLS. Correlation analysis between genes, body composition, and metabolic parameter of the human cohort were calculated using the R package ggstatsplot v0.9.185 [79] with the Spearman correlation coefficient and a confidence interval of 0.95. *p*-values were corrected for multiple inferences using the Holm method. Analyses were performed under R version 4.2.2 [80].

## Data Availability

The data presented in this study are available upon request from the corresponding author.

## Supplementary Materials

The following supporting information can be downloaded at: https://www.mdpi.com/article/10.3390/ijms24065672/s1.

## References

[1] Blüher M. (2019). Obesity: Global epidemiology and pathogenesis. Nat. Rev. Endocrinol..

[2] Johnston E.K., Abbott R.D. (2023). Adipose Tissue Paracrine-, Autocrine-, and Matrix-Dependent Signaling during the Development and Progression of Obesity. Cells.

[3] Sakers A., de Siqueira M.K., Seale P., Villanueva C.J. (2022). Adipose-tissue plasticity in health and disease. Cell.

[4] Datta R., Podolsky M.J., Atabai K. (2018). Fat fibrosis: Friend or foe?. JCI Insight.

[5] DeBari M.K., Abbott R.D. (2020). Adipose Tissue Fibrosis: Mechanisms, Models, and Importance. Int. J. Mol. Sci..

[6] Hirai S., Ohyane C., Kim Y.-I., Lin S., Goto T., Takahashi N., Kim C.-S., Kang J., Yu R., Kawada T. (2014). Involvement of mast cells in adipose tissue fibrosis. Am. J. Physiol. Endocrinol. Metab..

[7] Keophiphath M., Achard V., Henegar C., Rouault C., Clément K., Lacasa D. (2009). Macrophage-secreted factors promote a profibrotic phenotype in human preadipocytes. Mol. Endocrinol..

[8] Borthwick L.A., Wynn T.A., Fisher A.J. (2013). Cytokine mediated tissue fibrosis. Biochim. Biophys. Acta.

[9] Junttila I.S. (2018). Tuning the Cytokine Responses: An Update on Interleukin (IL)-4 and IL-13 Receptor Complexes. Front. Immunol..

[10] Zhu J. (2015). T helper 2 (Th2) cell differentiation, type 2 innate lymphoid cell (ILC2) development and regulation of interleukin-4 (IL-4) and IL-13 production. Cytokine.

[11] Zurawski S.M., Vega F., Huyghe B., Zurawski G. (1993). Receptors for interleukin-13 and interleukin-4 are complex and share a novel component that functions in signal transduction. EMBO J..

[12] Fichtner-Feigl S., Strober W., Kawakami K., Puri R.K., Kitani A. (2006). IL-13 signaling through the IL-13alpha2 receptor is involved in induction of TGF-beta1 production and fibrosis. Nat. Med..

[13] Wood N., Whitters M.J., Jacobson B.A., Witek J., Sypek J.P., Kasaian M., Eppihimer M.J., Unger M., Tanaka T., Goldman S.J. (2003). Enhanced interleukin (IL)-13 responses in mice lacking IL-13 receptor alpha 2. J. Exp. Med..

[14] Biernacka A., Dobaczewski M., Frangogiannis N.G. (2011). TGF-β signaling in fibrosis. Growth Factors.

[15] Marcelin G., Ferreira A., Liu Y., Atlan M., Aron-Wisnewsky J., Pelloux V., Botbol Y., Ambrosini M., Fradet M., Rouault C. (2017). A PDGFRα-Mediated Switch toward CD9high Adipocyte Progenitors Controls Obesity-Induced Adipose Tissue Fibrosis. Cell. Metab..

[16] Cinti S., Mitchell G., Barbatelli G., Murano I., Ceresi E., Faloia E., Wang S., Fortier M., Greenberg A.S., Obin M.S. (2005). Adipocyte death defines macrophage localization and function in adipose tissue of obese mice and humans. J. Lipid Res..

[17] Lindhorst A., Raulien N., Wieghofer P., Eilers J., Rossi F.M.V., Bechmann I., Gericke M. (2021). Adipocyte death triggers a pro-inflammatory response and induces metabolic activation of resident macrophages. Cell. Death Dis..

[18] Marcelin G., Gautier E.L., Clément K. (2022). Adipose Tissue Fibrosis in Obesity: Etiology and Challenges. Annu. Rev. Physiol..

[19] Jaitin D.A., Adlung L., Thaiss C.A., Weiner A., Li B., Descamps H., Lundgren P., Bleriot C., Liu Z., Deczkowska A. (2019). Lipid-Associated Macrophages Control Metabolic Homeostasis in a Trem2-Dependent Manner. Cell.

[20] Tanaka M., Ikeda K., Suganami T., Komiya C., Ochi K., Shirakawa I., Hamaguchi M., Nishimura S., Manabe I., Matsuda T. (2014). Macrophage-inducible C-type lectin underlies obesity-induced adipose tissue fibrosis. Nat. Commun..

[21] Vila I.K., Badin P.-M., Marques M.-A., Monbrun L., Lefort C., Mir L., Louche K., Bourlier V., Roussel B., Gui P. (2014). Immune cell Toll-like receptor 4 mediates the development of obesity- and endotoxemia-associated adipose tissue fibrosis. Cell. Rep..

[22] Klingberg F., Hinz B., White E.S. (2013). The myofibroblast matrix: Implications for tissue repair and fibrosis. J. Pathol..

[23] Antar S.A., Ashour N.A., Marawan M.E., Al-Karmalawy A.A. (2023). Fibrosis: Types, Effects, Markers, Mechanisms for Disease Progression, and Its Relation with Oxidative Stress, Immunity, and Inflammation. Int. J. Mol. Sci..

[24] Chomarat P., Banchereau J. (1998). Interleukin-4 and interleukin-13: Their similarities and discrepancies. Int. Rev. Immunol..

[25] Van Rooijen N., van Nieuwmegen R. (1984). Elimination of phagocytic cells in the spleen after intravenous injection of liposome-encapsulated dichloromethylene diphosphonate. An enzyme-histochemical study. Cell. Tissue Res..

[26] Sun K., Tordjman J., Clément K., Scherer P.E. (2013). Fibrosis and adipose tissue dysfunction. Cell. Metab..

[27] Gericke M., Weyer U., Braune J., Bechmann I., Eilers J. (2015). A method for long-term live imaging of tissue macrophages in adipose tissue explants. Am. J. Physiol. Endocrinol. Metab..

[28] D’Arcy Q., Gharaee-Kermani M., Zhilin-Roth A., Macoska J.A. (2022). The IL-4/IL-13 signaling axis promotes prostatic fibrosis. PLoS ONE.

[29] Jinnin M., Ihn H., Yamane K., Tamaki K. (2004). Interleukin-13 stimulates the transcription of the human alpha2(I) collagen gene in human dermal fibroblasts. J. Biol. Chem..

[30] Kaviratne M., Hesse M., Leusink M., Cheever A.W., Davies S.J., McKerrow J.H., Wakefield L.M., Letterio J.J., Wynn T.A. (2004). IL-13 activates a mechanism of tissue fibrosis that is completely TGF-beta independent. J. Immunol..

[31] Aoudjehane L., Pissaia A., Scatton O., Podevin P., Massault P.-P., Chouzenoux S., Soubrane O., Calmus Y., Conti F. (2008). Interleukin-4 induces the activation and collagen production of cultured human intrahepatic fibroblasts via the STAT-6 pathway. Lab. Investig..

[32] Nguyen J.K., Austin E., Huang A., Mamalis A., Jagdeo J. (2020). The IL-4/IL-13 axis in skin fibrosis and scarring: Mechanistic concepts and therapeutic targets. Arch. Dermatol. Res..

[33] Lee C.G., Homer R.J., Zhu Z., Lanone S., Wang X., Koteliansky V., Shipley J.M., Gotwals P., Noble P., Chen Q. (2001). Interleukin-13 induces tissue fibrosis by selectively stimulating and activating transforming growth factor beta(1). J. Exp. Med..

[34] O’Brien J., Lyons T., Monks J., Lucia M.S., Wilson R.S., Hines L., Man Y.-g., Borges V., Schedin P. (2010). Alternatively activated macrophages and collagen remodeling characterize the postpartum involuting mammary gland across species. Am. J. Pathol..

[35] Takemoto R., Kamiya T., Atobe T., Hara H., Adachi T. (2021). Regulation of lysyl oxidase expression in THP-1 cell-derived M2-like macrophages. J. Cell. Biochem..

[36] Da Costa Santos M.A.R., Dos Reis J.S., do Nascimento Santos C.A., da Costa K.M., Barcelos P.M., de Oliveira Francisco K.Q., Barbosa P.A.G.N., da Silva E.D.S., Freire-de-Lima C.G., Morrot A. (2023). Expression of O-glycosylated oncofetal fibronectin in alternatively activated human macrophages. Immunol. Res..

[37] Nakamura R., Bing R., Gartling G.J., Branski R.C. (2022). Macrophages alter inflammatory and fibrotic gene expression in human vocal fold fibroblasts. Exp. Cell. Res..

[38] Rudnik M., Hukara A., Kocherova I., Jordan S., Schniering J., Milleret V., Ehrbar M., Klingel K., Feghali-Bostwick C., Distler O. (2021). Elevated Fibronectin Levels in Profibrotic CD14+ Monocytes and CD14+ Macrophages in Systemic Sclerosis. Front. Immunol..

[39] Munder M., Eichmann K., Morán J.M., Centeno F., Soler G., Modolell M. (1999). Th1/Th2-regulated expression of arginase isoforms in murine macrophages and dendritic cells. J. Immunol..

[40] Lindemann D., Racké K. (2003). Glucocorticoid inhibition of interleukin-4 (IL-4) and interleukin-13 (IL-13) induced up-regulation of arginase in rat airway fibroblasts. Naunyn Schmiedebergs Arch. Pharmacol..

[41] Haase J., Weyer U., Immig K., Klöting N., Blüher M., Eilers J., Bechmann I. (2014). Local proliferation of macrophages in adipose tissue during obesity-induced inflammation. Diabetologia.

[42] Ichioka M., Suganami T., Tsuda N., Shirakawa I., Hirata Y., Satoh-Asahara N., Shimoda Y., Tanaka M., Kim-Saijo M., Miyamoto Y. (2011). Increased expression of macrophage-inducible C-type lectin in adipose tissue of obese mice and humans. Diabetes.

[43] Ramachandran P., Dobie R., Wilson-Kanamori J.R., Dora E.F., Henderson B.E.P., Luu N.T., Portman J.R., Matchett K.P., Brice M., Marwick J.A. (2019). Resolving the fibrotic niche of human liver cirrhosis at single-cell level. Nature.

[44] Itoh M., Kato H., Suganami T., Konuma K., Marumoto Y., Terai S., Sakugawa H., Kanai S., Hamaguchi M., Fukaishi T. (2013). Hepatic crown-like structure: A unique histological feature in non-alcoholic steatohepatitis in mice and humans. PLoS ONE.

[45] Marangoni R.G., Korman B.D., Wei J., Wood T.A., Graham L.V., Whitfield M.L., Scherer P.E., Tourtellotte W.G., Varga J. (2015). Myofibroblasts in murine cutaneous fibrosis originate from adiponectin-positive intradermal progenitors. Arthritis Rheumatol..

[46] Jones J.E.C., Rabhi N., Orofino J., Gamini R., Perissi V., Vernochet C., Farmer S.R. (2020). The Adipocyte Acquires a Fibroblast-Like Transcriptional Signature in Response to a High Fat Diet. Sci. Rep..

[47] Marangoni R.G., Korman B., Varga J. (2020). Adipocytic Progenitor Cells Give Rise to Pathogenic Myofibroblasts: Adipocyte-to-Mesenchymal Transition and Its Emerging Role in Fibrosis in Multiple Organs. Curr. Rheumatol. Rep..

[48] Côté J.A., Lessard J., Pelletier M., Marceau S., Lescelleur O., Fradette J., Tchernof A. (2017). Role of the TGF-β pathway in dedifferentiation of human mature adipocytes. FEBS Open Bio.

[49] Tsao C.-H., Shiau M.-Y., Chuang P.-H., Chang Y.-H., Hwang J. (2014). Interleukin-4 regulates lipid metabolism by inhibiting adipogenesis and promoting lipolysis. J. Lipid Res..

[50] Spencer M., Yao-Borengasser A., Unal R., Rasouli N., Gurley C.M., Zhu B., Peterson C.A., Kern P.A. (2010). Adipose tissue macrophages in insulin-resistant subjects are associated with collagen VI and fibrosis and demonstrate alternative activation. Am. J. Physiol. Endocrinol. Metab..

[51] Sarsenbayeva A., Pereira M.J., Nandi Jui B., Ahmed F., Dipta P., Fanni G., Almby K., Kristófi R., Hetty S., Eriksson J.W. (2022). Excess glucocorticoid exposure contributes to adipose tissue fibrosis which involves macrophage interaction with adipose precursor cells. Biochem. Pharmacol..

[52] Coutinho H.M., Acosta L.P., Wu H.W., McGarvey S.T., Su L., Langdon G.C., Jiz M.A., Jarilla B., Olveda R.M., Friedman J.F. (2007). Th2 cytokines are associated with persistent hepatic fibrosis in human *Schistosoma japonicum* infection. J. Infect. Dis..

[53] Chandriani S., DePianto D.J., N’Diaye E.N., Abbas A.R., Jackman J., Bevers J., Ramirez-Carrozzi V., Pappu R., Kauder S.E., Toy K. (2014). Endogenously expressed IL-13Rα2 attenuates IL-13-mediated responses but does not activate signaling in human lung fibroblasts. J. Immunol..

[54] Kwon H., Laurent S., Tang Y., Zong H., Vemulapalli P., Pessin J.E. (2014). Adipocyte-specific IKKβ signaling suppresses adipose tissue inflammation through an IL-13-dependent paracrine feedback pathway. Cell Rep..

[55] El-Wakkad A., Hassan N.E.-M., Sibaii H., El-Zayat S.R. (2013). Proinflammatory, anti-inflammatory cytokines and adiponkines in students with central obesity. Cytokine.

[56] Chang Y.-H., Ho K.-T., Lu S.-H., Huang C.-N., Shiau M.-Y. (2012). Regulation of glucose/lipid metabolism and insulin sensitivity by interleukin-4. Int. J. Obes. (Lond.).

[57] Pasarica M., Gowronska-Kozak B., Burk D., Remedios I., Hymel D., Gimble J., Ravussin E., Bray G.A., Smith S.R. (2009). Adipose tissue collagen VI in obesity. J. Clin. Endocrinol. Metab..

[58] Khan T., Muise E.S., Iyengar P., Wang Z.V., Chandalia M., Abate N., Zhang B.B., Bonaldo P., Chua S., Scherer P.E. (2009). Metabolic dysregulation and adipose tissue fibrosis: Role of collagen VI. Mol. Cell. Biol..

[59] Braune J., Weyer U., Hobusch C., Mauer J., Brüning J.C., Bechmann I., Gericke M. (2017). IL-6 Regulates M2 Polarization and Local Proliferation of Adipose Tissue Macrophages in Obesity. J. Immunol..

[60] Van Rooijen N., Sanders A., van den Berg T.K. (1996). Apoptosis of macrophages induced by liposome-mediated intracellular delivery of clodronate and propamidine. J. Immunol. Methods.

[61] Ackermann J., Arndt L., Kirstein M., Hobusch C., Brinker G., Klöting N., Braune J., Gericke M. (2021). Myeloid Cell-Specific IL-4 Receptor Knockout Partially Protects from Adipose Tissue Inflammation. J. Immunol..

[62] Braune J., Lindhorst A., Fröba J., Hobusch C., Kovacs P., Blüher M., Eilers J., Bechmann I., Gericke M. (2021). Multinucleated Giant Cells in Adipose Tissue Are Specialized in Adipocyte Degradation. Diabetes.

[63] Brentnall M., Weir D.B., Rongvaux A., Marcus A.I., Boise L.H. (2014). Procaspase-3 regulates fibronectin secretion and influences adhesion, migration and survival independently of catalytic function. J. Cell. Sci..

[64] Brinker G., Froeba J., Arndt L., Braune J., Hobusch C., Lindhorst A., Bechmann I., Gericke M. (2021). CD^4+^ T cells regulate glucose homeostasis independent of adipose tissue dysfunction in mice. Eur. J. Immunol..

[65] Hashimshony T., Senderovich N., Avital G., Klochendler A., de Leeuw Y., Anavy L., Gennert D., Li S., Livak K.J., Rozenblatt-Rosen O. (2016). CEL-Seq2: Sensitive highly-multiplexed single-cell RNA-Seq. Genome Biol..

[66] Martin M. (2011). Cutadapt removes adapter sequences from high-throughput sequencing reads. EMBnet J..

[67] Kim D., Langmead B., Salzberg S.L. (2015). HISAT: A fast spliced aligner with low memory requirements. Nat. Methods.

[68] Li H., Handsaker B., Wysoker A., Fennell T., Ruan J., Homer N., Marth G., Abecasis G., Durbin R. (2009). The Sequence Alignment/Map format and SAMtools. Bioinformatics.

[69] Liao Y., Smyth G.K., Shi W. (2014). featureCounts: An efficient general purpose program for assigning sequence reads to genomic features. Bioinformatics.

[70] Aken B.L., Achuthan P., Akanni W., Amode M.R., Bernsdorff F., Bhai J., Billis K., Carvalho-Silva D., Cummins C., Clapham P. (2017). Ensembl 2017. Nucleic Acids Res..

[71] Robinson M.D., McCarthy D.J., Smyth G.K. (2010). edgeR: A Bioconductor package for differential expression analysis of digital gene expression data. Bioinformatics.

[72] Robinson M.D., Oshlack A. (2010). A scaling normalization method for differential expression analysis of RNA-seq data. Genome Biol..

[73] Langhardt J., Flehmig G., Klöting N., Lehmann S., Ebert T., Kern M., Schön M.R., Gärtner D., Lohmann T., Dressler M. (2018). Effects of Weight Loss on Glutathione Peroxidase 3 Serum Concentrations and Adipose Tissue Expression in Human Obesity. Obes. Facts.

[74] Klöting N., Blüher M. (2010). Insulin-sensitive obesity. Am. J. Physiol. Endocrinol. Metab..

[75] Picelli S., Faridani O.R., Björklund A.K., Winberg G., Sagasser S., Sandberg R. (2014). Full-length RNA-seq from single cells using Smart-seq2. Nat. Protoc..

[76] Chen S., Zhou Y., Chen Y., Gu J. (2018). fastp: An ultra-fast all-in-one FASTQ preprocessor. Bioinformatics.

[77] Bray N.L., Pimentel H., Melsted P., Pachter L. (2016). Near-optimal probabilistic RNA-seq quantification. Nat. Biotechnol..

[78] Love M.I., Huber W., Anders S. (2014). Moderated estimation of fold change and dispersion for RNA-seq data with DESeq2. Genome Biol..

[79] Patil I. (2021). Visualizations with statistical details: The ‘ggstatsplot’ approach. JOSS.

[80] R Foundation for Statistical Computing (2022). R Core Team R: A Language and Environment for Statistical Computing. http://www.r-project.org.

