# Supplementary material for: The Role of IL-13 and IL-4 in Adipose Tissue Fibrosis
Source: Int J Mol Sci. 2023 Mar 16;24(6):5672. doi: 10.3390/ijms24065672 (PMC10051142; doi:10.3390/ijms24065672)
Supplement: Supplementary file 1 [file ijms-24-05672-s001.zip › ijms-2282264-supplementary.pdf]

Supplementary Material

The role of IL-13 and IL-4 in adipose tissue fibrosis

Lilli Arndt [1,2], Andreas Lindhorst [1], Julia Neugebauer [1], Anne Hoffmann [3], Constance Hobusch [1], Vasileia-Ismini Alexaki [4], Adhideb Ghosh [5,6], Matthias Blüher [3], Christian Wolfrum [5], Markus Glaß [7] and Martin Gericke [1,2,*]

[1] Institute of Anatomy, Leipzig University, 04103 Leipzig, Germany
[2] Institute of Anatomy and Cell Biology, Martin-Luther-University Halle-Wittenberg, 06108 Halle (Saale), Germany
[3] Helmholtz Institute for Metabolic, Obesity and Vascular Research, 04103 Leipzig, Germany
[4] Institute of Clinical Chemistry and Laboratory Medicine, University Hospital Dresden, 01307 Dresden, Germany
[5] Institute of Food, Nutrition and Health, ETH Zurich, 8603 Schwerzenbach, Switzerland
[6] Functional Genomics Center Zurich, ETH Zurich and University of Zurich, 8057 Zurich, Switzerland
[7] Institute of Molecular Medicine, Martin-Luther-University Halle-Wittenberg, 06120 Halle (Saale), Germany
* Correspondence: martin.gericke@medizin.uni-leipzig.de; Tel.: +49-3431-22055

Supplementary Figures

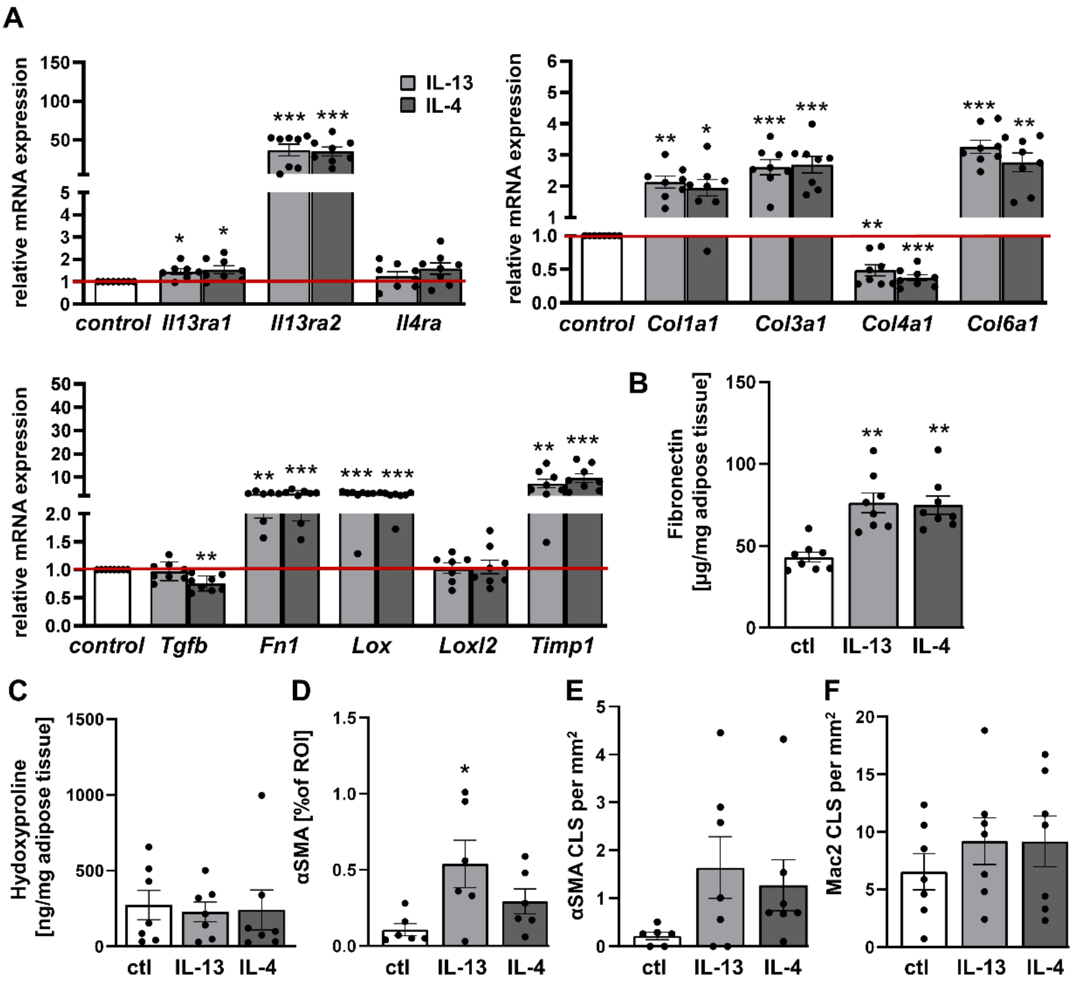

Figure S1: Fibrosis data in B6J mice.

Adipose tissue explants from B6J mice (n=8) were stimulated either with IL-13 (50 ng/ml) or IL-4 (50 ng/ml) or non-stimulated (control) for four days. (**A**) mRNA expression of cytokine receptors and fibrosis markers are given as fold change compared to the control condition setting to 1. (**B**) Levels of fibronectin were determined in supernatants of the explants (n=8) by ELISA. (**C**) Content of hydroxyproline was measured in AT explants by hydroxyproline assay (n=7). The αSMA positive area (**D**) and the number of αSMA positive CLS (**E**) and Mac-2 positive CLS (**F**) were quantified by immunostainings. Data represented as mean ± SEM. *p-value <0.05; **p-value <0.01; ***p-value <0.001.

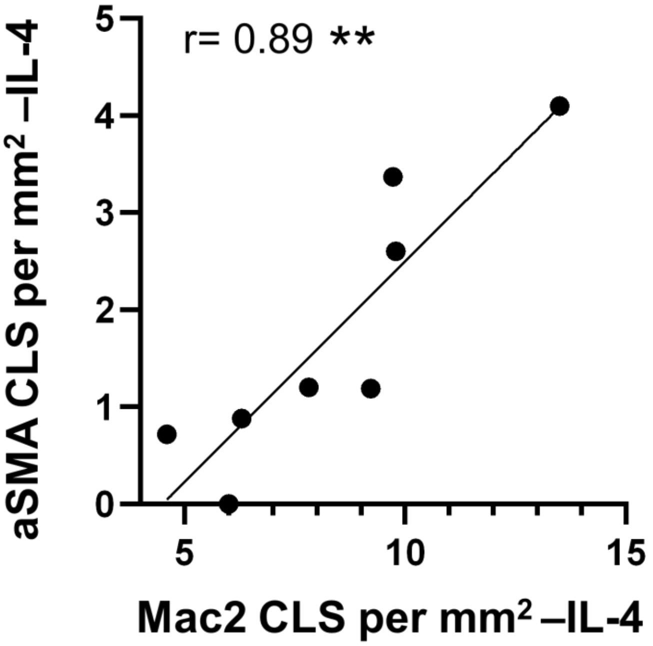

Figure S2: Correlation of aSMA-positive CLS with Mac2-positive CLS in C3H mice. WAT explants from C3H mice were stimulated with IL-4 (50ng/ml) four days and immunofluorescence staining was performed with antibodies against α-SMA and Mac2. The number of α-SMA positive CLS were correlated with the number of Mac2 positive CLS (n=8). For correlation analysis a Pearson correlation was used; **p-value <0.01.

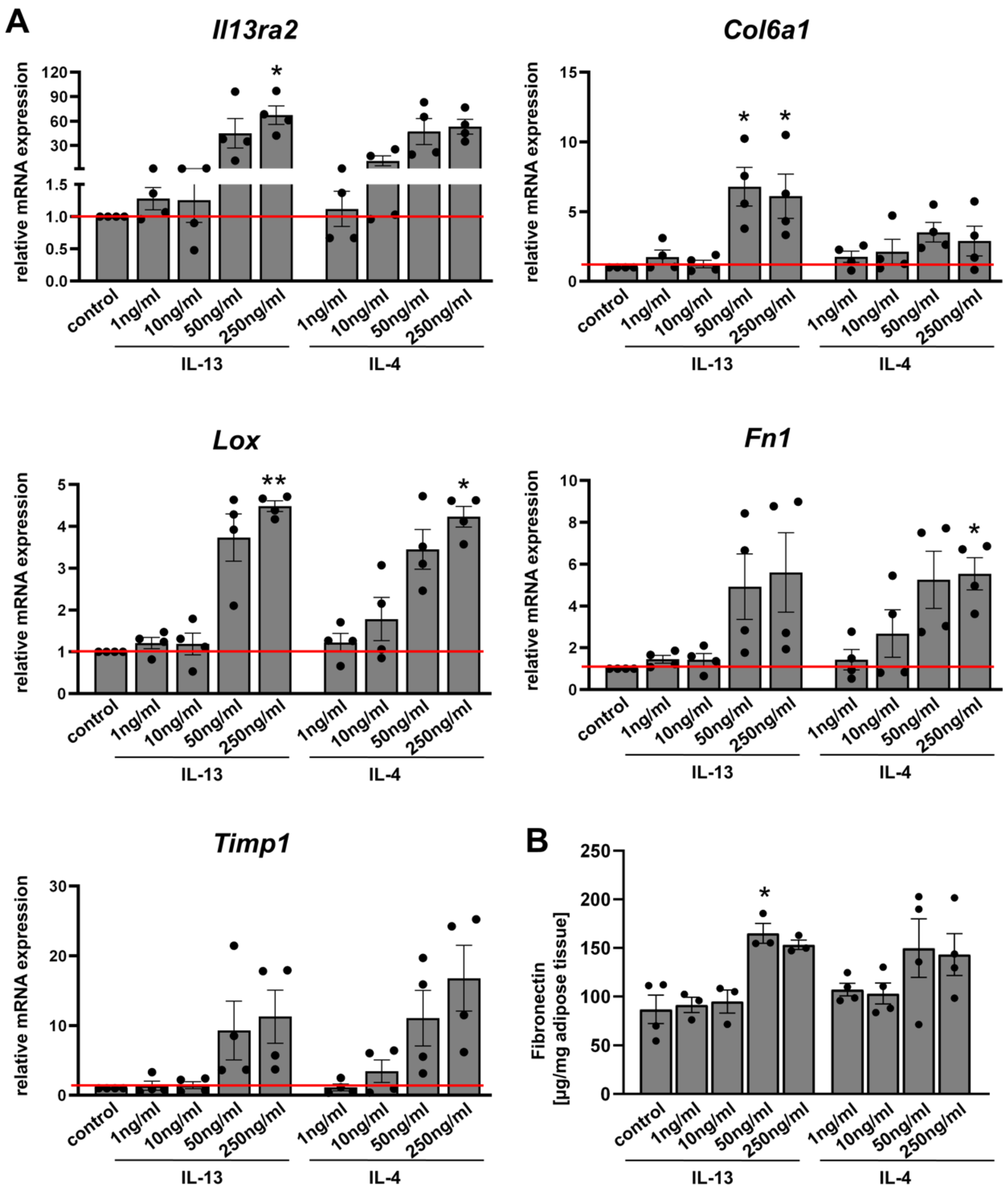

Figure S3: Concentration series of IL-13 and IL-4 in B6J mice.

WAT explants from B6J mice (n=4) were stimulated with increasing concentrations of IL-13 or IL-4 (1 ng/ml up to 250 ng/ml) or non-stimulated (control) for four days. (**A**) Fibrosis markers were determined by qPCR (stimulated data are represented as fold change compared to control data) (**B**) and fibronectin levels in the supernatants by ELISA. Data represented as mean ± SEM. *p-value <0.05; **p-value <0.01.

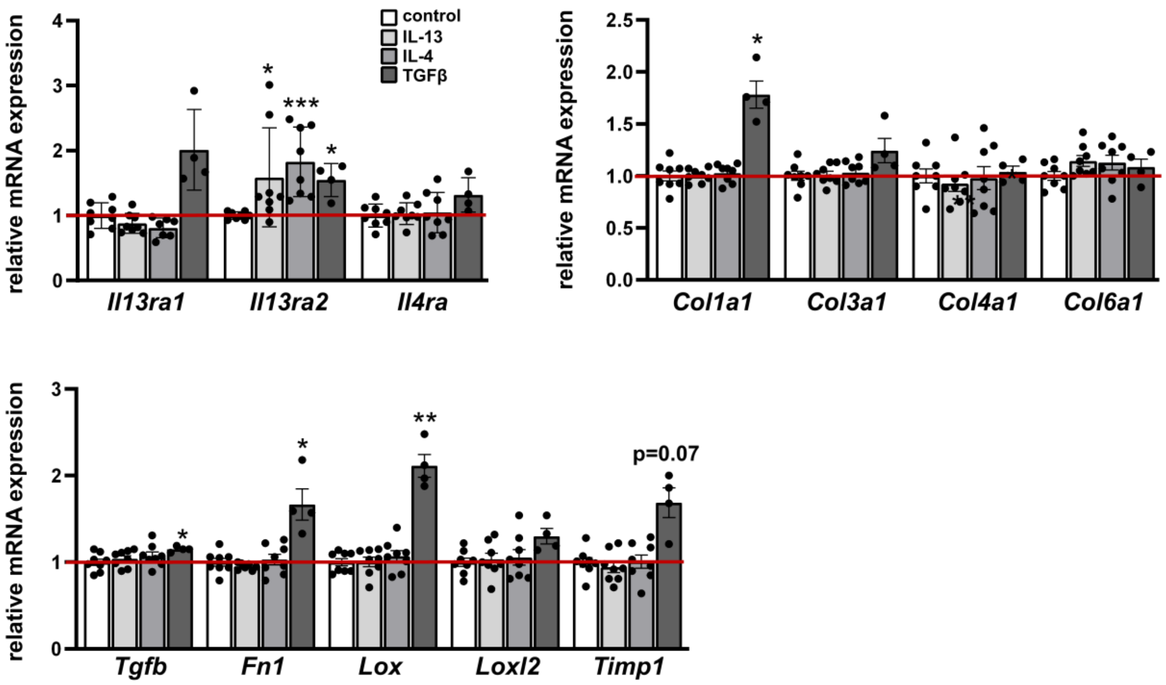

Figure S4: Fibrosis marker expression in 3T3-L1 differentiated adipocytes.

3T3-L1 cells were differentiated into adipocytes and stimulated with IL-13 or IL-4 (50 ng/ml) or Tgf-β1 (5 ng/ml) for 48h. mRNA expression was measured by qPCR and calculated as fold change compared to non-stimulated adipocytes (control). Data are presented as mean + SEM of three or four independent experiments with repeated approaches (n=4-8); *p-value <0.05; **p-value <0.01; ***p-value <0.001.

subcutanous adipose tissue

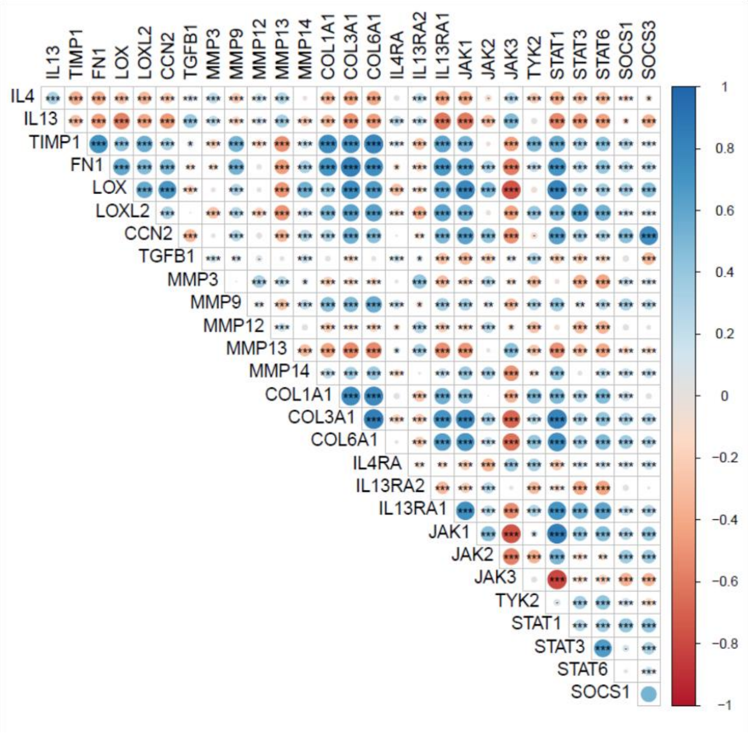

Figure S5: Gene correlation analysis of human subcutaneous adipose tissues. The presented data are RNA-Seq data from human subcutaneous adipose tissue samples (n=1,553). Correlation analysis of fibrotic-related genes as well as *IL-13* and *IL-4*. Positive correlations are shown in blue, while negative correlations are represented in red. The size of the dot refers to the degree of correlation; *p-value <0.05; **p-value <0.01; ***p-value <0.001.

Supplementary Tables

Table S1: Mouse primer sequences for quantitative RT-PCR.

**Primer**	**Sequenz 5' -> 3'**
Ipo8_for	ACAAGCTCTGCTGACTGTGC
Ipo8_rev	CAGTGTCCTTCGGTGCTCTG
IL4Ra_for	GAGGGACCTGGCTTCTGATT
IL4Ra_rev	CCTTGATGCTCCCAGATCCA
IL13Ra1_for	GGTGGGCTCTCAGTGTAGTG
IL13Ra1_rev	TCAGGATCACCTTCAGGGGG
IL13Ra2_for	ACACAGGGCCAGACTCAAAG
IL13Ra2_rev	TGGAGGCTCAATGTGGGTTC
Tgfb_for	CTGCTGACCCCCACTGATAC
Tgfb_for	AGCCCTGTATTCCGTCTCCT
Itgam (Cd11b)_for	ATGGACGCTGATGGCAATACC
Itgam (Cd11b)_rev	TCCCCATTCACGTCTCCCA
Col1a1_for	CGATGGATTCCCGTTCGAGT
Col1a1_rev	GAGGCCTCGGTGGACATTAG
Col3a1_for	CTGTAACATGGAAACTGGGGAAA
Col3a1_rev	CCATAGCTGAACTGAAAACCACC
Col4a1_for	CTGGCACAAAAGGGACGAG
Col4a1_rev	ACGTGGCCGAGAATTTCACC
Col6a1_for	CTGCTGCTACAAGCCTGCT
Col6a1_rev	CCCCATAAGGTTTCAGCCTCA
Lox_for	CAGCCACATAGATCGCATGGT
Lox_rev	GCCGTATCCAGGTCGGTTC
Loxl2_for	ATTAACCCCAACTATGAAGTGCC
Loxl2_rev	CTGTCTCCTCACTGAAGGCTC
Fn1_for	TTCAAGTGTGATCCCCATGAAG
Fn1_rev	CAGGTCTACGGCAGTTGTCA
Timp1_for	CGAGACCACCTTATACCAGCG
Timp1_rev	ATGACTGGGGTGTAGGCGTA

Table S2: Correlation values in visceral adipose tissue from genes with metabolic data

Phenotypes	Corr IL4	adj. P-value IL4	Code IL4	Corr IL13	adj. P-value IL13	Code IL13	Corr TIMP1	adj. P-value TIMP1	Code TIMP1	Corr FN1	adj. P-value FN1	Code FN1
NEFA	0.1901	1		0.02638	1		0.1675	1		0.1985	1	
Cholesterol	0.08597	0.095084		0.02357	1		-0.07175	0.36463		-0.003369	1	
LDL Cholesterol	0.05061	1		-0.001516	1		-0.01179	1		0.03894	1	
HbA1c	0.0403	1		0.009805	1		-0.05019	1		-0.00368	1	
Diabetes	0.0306	1		0.02955	1		-0.001653	1		0.01459	1	
HDL Cholesterol	0.02614	1		0.008203	1		-0.008739	1		-0.03501	1	
FPG	0.02407	1		0.02578	1		-0.01755	1		-0.01529	1	
Age	0.01957	1		0.03187	1		-0.01645	1		-0.02385	1	
CrP	0.01356	1		0.04666	0.84633		-0.02405	1		-0.03569	1	
Height	-0.003776	1		-0.01084	1		0.01761	1		0.0219	1	
WHR	-0.01794	1		-0.02922	1		-0.02715	1		0.07791	1	
Body weight	-0.03605	1		-0.08069	0.027695	*	0.03554	1		0.06076	0.26639	
BMI	-0.03733	1		-0.07005	0.092021		0.03681	1		0.05901	0.28066	
Leptin	-0.05239	1		-0.08988	0.42081		0.1123	0.1335		0.0773	0.85619	
Adiponectin	-0.06606	1		0.02474	1		0.2379	0.18369		0.003568	1	
Body fat	-0.1069	0.07685	.	-0.1375	0.0050682	**	0.2537	$2.24x10^{-10}$	***	0.2044	$1.06X10^{-6}$	***
FPI	-0.1594	0.007561	**	-0.1178	0.14009		0.02904	1		0.1316	0.058568	
HOMA-IR	-0.1683	0.0069919	**	-0.1232	0.14009		0.02996	1		0.1501	0.025751	*
Waist	-0.1949	0.015365	*	-0.2693	$6.99x10^{-5}$	***	0.1255	0.50287		0.2546	0.00022736	***

Phenotypes	Corr LOX	adj. P-value LOX	Code LOX	Corr LOXL2	adj. P-value LOXL2	Code LOXL2	Corr CCN2	adj. P-value CCN2	Code CCN2	Corr TGFB1	adj. P-value TGFB1	Code TGFB1
NEFA	0.139	1		0.1332	1		0.1563	1		-0.04774	1	
Cholesterol	0.01239	1		-0.03954	1		0.02546	1		0.002711	1	
LDL Cholesterol	0.03906	1		-0.04648	1		0.03201	1		0.00747	1	
HbA1c	0.008841	1		0.1021	0.046455	*	-0.006924	1		-0.03881	1	
Diabetes	-0.01522	1		0.06099	0.21691		0.02715	1		-0.04049	1	
HDL Cholesterol	-0.07729	0.25522		-0.0767	0.21691		-0.04246	1		0.01562	1	
FPG	-0.01454	1		0.1054	0.0013492	**	-0.008076	1		0.019	1	
Age	-0.01628	1		-0.02027	1		-0.01414	1		0.03125	1	
CrP	-0.04329	0.93383		0.02558	1		-0.02254	1		0.0437	1	
Height	0.02728	1		0.01811	1		-0.006735	1		0.006785	1	
WHR	0.01268	1		0.0615	1		0.05263	1		0.08595	1	
Body weight	0.05334	0.48702		0.03638	1		0.0436	1		-0.04534	1	
BMI	0.05048	0.51384		0.03212	1		0.05505	0.42087		-0.04098	1	
Leptin	0.1333	0.024848	*	0.1145	0.088911	.	0.1325	0.028151	*	-0.01984	1	
Adiponectin	0.1936	0.48702		0.102	1		-0.00064	1		0.07075	1	
Body fat	0.1232	0.020657	*	0.07541	0.5177		0.09617	0.18006		0.04846	1	
FPI	0.09835	0.42232		0.205	$9.36x10^{-5}$	***	0.1064	0.28529		-0.0423	1	
HOMA-IR	0.1202	0.17953		0.2433	$3.97x10^{-6}$	***	0.1218	0.1737		-0.04802	1	
Waist	0.2414	0.0006787	***	0.1752	0.046455	*	0.2761	$3.87x10^{-5}$	***	-0.1562	0.1526	

Phenotypes	Corr MMP3	adj. P-value MMP3	Code MMP3	Corr MMP9	adj. P-value MMP9	Code MMP9	Corr MMP12	adj. P-value MMP12	Code MMP12	Corr MMP13	adj. P-value MMP13	Code MMP13
NEFA	-0.1457	1		0.08109	1		0.05823	1		0.1813	1	
Cholesterol	0.01063	1		-0.01768	1		0.04069	1		0.07175	0.38732	
LDL Cholesterol	-0.01667	1		-0.004943	1		0.01071	1		0.03583	1	
HbA1c	0.03548	1		0.03447	1		0.07308	0.50219		0.04441	1	
Diabetes	0.01093	1		-0.001574	1		0.07099	0.10101		0.04202	1	
HDL Cholesterol	0.03071	1		-0.07614	0.33709		-0.01302	1		-0.001339	1	
FPG	-0.007209	1		0.02281	1		0.05829	0.49911		0.02513	1	
Age	-0.0009319	1		0.006714	1		-0.01154	1		0.004115	1	
CrP	0.04543	1		0.07855	0.043836	*	0.05597	0.49911		0.04332	1	
Height	0.02303	1		0.01901	1		-0.02095	1		-0.001581	1	
WHR	0.06714	1		0.06017	1		-0.01888	1		0.02846	1	
Body weight	-0.004807	1		-0.007866	1		-0.02694	1		-0.02581	1	
BMI	-0.02418	1		-0.004904	1		-0.01329	1		-0.03656	1	
Leptin	-0.03934	1		-0.01071	1		-0.03045	1		-0.02443	1	
Adiponectin	-0.1629	1		-0.06671	1		-0.2195	0.34089		-0.3396	0.0041624	**
Body fat	-0.0755	0.88891		-0.05313	1		-0.08073	0.50219		-0.09519	0.2181	
FPI	-0.0492	1		0.04348	1		0.009973	1		-0.03949	1	
HOMA-IR	-0.08024	1		0.07578	1		0.02065	1		-0.05691	1	
Waist	-0.01876	1		0.007975	1		-0.09002	1		-0.00491	1	

Phenotypes	Corr MMP14	adj. P-value MMP14	Code MMP14	Corr COL1A1	adj. P-value COL1A1	Code COL1A1	Corr COL3A1	adj. P-value COL3A1	Code COL3A1	Corr COL6A1	adj. P-value COL6A1	Code COL6A1
NEFA	0.1084	1		0.01466	1		0.09862	1		0.07433	1	
Cholesterol	0.03642	1		-0.07904	0.19363		-0.04062	1		-0.04787	1	
LDL Cholesterol	0.02662	1		-0.009319	1		0.009198	1		-0.007326	1	
HbA1c	0.01349	1		-0.04693	1		-0.006616	1		-0.02244	1	
Diabetes	-0.0151	1		-0.01836	1		0.02883	1		-0.01246	1	
HDL Cholesterol	0.02099	1		0.01975	1		-0.03625	1		0.00177	1	
FPG	-0.01398	1		-0.03417	1		-0.008487	1		0.002926	1	
Age	0.03095	1		-0.01943	1		-0.00608	1		-0.01092	1	
CrP	-0.01405	1		-0.02101	1		-0.04034	1		-0.02585	1	
Height	0.007906	1		0.01972	1		0.008899	1		0.01758	1	
WHR	0.0376	1		-0.005308	1		-0.001885	1		0.01918	1	
Body weight	-0.03855	1		0.03502	1		0.03398	1		0.02929	1	
BMI	-0.04481	1		0.0378	1		0.04401	1		0.0316	1	
Leptin	-0.09944	0.26786		0.1078	0.17408		0.1071	0.18235		0.08015	0.84716	
Adiponectin	0.2426	0.15819		0.3119	0.013131	*	0.1685	1		0.2881	0.033835	*
Body fat	0.02018	1		0.2174	$1.37x10^{-7}$	***	0.1625	0.00032383	***	0.1864	$1.45x10^{-5}$	***
FPI	-0.1337	0.056569		0.05359	1		0.09151	0.6564		0.07786	1	
HOMA-IR	-0.1153	0.24071		0.05892	1		0.1198	0.1849		0.1024	0.5261	
Waist	-0.2605	0.00014733	***	0.07189	1		0.1918	0.01963	*	0.1164	0.78074	

Phenotypes	Corr IL4RA	adj. P-value IL4RA	Code IL4RA	Corr IL13RA2	adj. P-value IL13RA2	Code IL13RA2	Corr IL13RA1	adj. P-value IL13RA1	Code IL13RA1	Corr JAK1	adj. P-value JAK1	Code JAK1	Corr JAK2	adj. P-value JAK2	Code JAK2
NEFA	0.1092	1		-0.1279	1		0.06962	1		0.1049	1		0.258	0.46801	
Cholesterol	-0.02213	1		0.03548	1		-0.0602	0.73014		-0.03368	1		0.04171	1	
LDL Cholesterol	0.0006553	1		0.01213	1		-0.02428	1		-0.007244	1		0.01376	1	
HbA1c	-0.04511	1		0.04447	1		-0.0603	0.88966		-0.0426	1		0.03124	1	
Diabetes	0.005399	1		0.05903	0.39006		0.005373	1		-0.0251	1		0.02589	1	
HDL Cholesterol	-0.0119	1		0.04491	1		-0.05209	1		-0.02958	1		-0.07442	0.38863	
FPG	0.009098	1		0.04007	1		-0.03486	1		-0.03517	1		0.006913	1	
Age	0.04114	1		0.02521	1		-0.02907	1		-0.01161	1		-0.0133	1	
CrP	0.0235	1		0.01417	1		-0.06776	0.15473		-0.06626	0.17318		-0.01774	1	
Height	-0.001799	1		0.002723	1		0.02369	1		0.01345	1		0.02402	1	
WHR	0.0002111	1		-0.06743	1		0.0222	1		0.01794	1		0.1526	0.86484	
Body weight	-0.0179	1		-0.007473	1		0.05232	0.57779		0.03727	1		0.02465	1	
BMI	-0.009851	1		-0.006157	1		0.046	0.83956		0.04107	1		0.01603	1	
Leptin	-0.02092	1		-0.0456	1		0.03719	1		0.04613	1		-0.02479	1	
Adiponectin	0.1011	1		0.09534	1		0.1306	1		0.2585	0.098716		-0.1473	1	
Body fat	-0.0483	1		0.0002013	1		0.08707	0.34877		0.1273	0.014721	*	-0.001557	1	
FPI	-0.05427	1		0.0004188	1		0.09797	0.46236		0.06466	1		0.04092	1	
HOMA-IR	-0.01597	1		-0.006387	1		0.1219	0.17215		0.09592	0.6507		0.04706	1	
Waist	-0.09384	1		-0.07335	1		0.1888	0.024956	*	0.1467	0.20592		0.1748	0.056202	

Phenotypes	Corr IL4RA	adj. P-value IL4RA	Corr JAK3	adj. P-value JAK3	Code JAK3	Corr TYK2	adj. P-value TYK2	Code TYK2	Corr STAT1	adj. P-value STAT1	Code STAT1	Corr STAT3	adj. P-value STAT3	Code STAT3
NEFA	0.1092	1	-0.07272	1		0.007408	1		0.1666	1		0.05808	1	
Cholesterol	-0.02213	1	-0.02869	1		-0.05532	1		-0.05613	0.97524		-0.04624	1	
LDL Cholesterol	0.0006553	1	-0.02726	1		-0.01332	1		-0.03889	1		-0.033	1	
HbA1c	-0.04511	1	0.02913	1		-0.08164	0.34247		-0.03755	1		-0.01291	1	
Diabetes	0.005399	1	-0.01201	1		-0.04776	0.97539		7.32e-05	1		0.04389	1	
HDL Cholesterol	-0.0119	1	0.02816	1		0.005008	1		-0.07635	0.33133		-0.06182	0.84643	
FPG	0.009098	1	0.002226	1		-0.01433	1		-0.02324	1		0.04929	0.91742	
Age	0.04114	1	0.02759	1		-0.01451	1		-0.004999	1		-0.01349	1	
CrP	0.0235	1	0.08154	0.02804	*	-0.02218	1		-0.05613	0.44329		-0.03448	1	
Height	-0.001799	1	0.005229	1		0.01222	1		0.02433	1		-0.008981	1	
WHR	0.0002111	1	-0.01685	1		-0.09146	1		0.02013	1		-0.0283	1	
Body weight	-0.0179	1	-0.02562	1		0.008068	1		0.03885	1		0.02248	1	
BMI	-0.009851	1	-0.04056	1		0.01275	1		0.04055	1		0.02409	1	
Leptin	-0.02092	1	-0.03442	1		0.03457	1		0.04292	1		0.05474	1	
Adiponectin	0.1011	1	-0.1805	0.77973		0.1795	0.95108		0.1931	0.55411		0.08096	1	
Body fat	-0.0483	1	-0.2088	5.37×10^{-7}	***	0.0742	0.91313		0.08896	0.33133		0.1465	0.0020438	**
FPI	-0.05427	1	-0.09567	0.5599		0.04173	1		0.07471	1		0.09524	0.57301	
HOMA-IR	-0.01597	1	-0.09229	0.77973		0.07197	1		0.11	0.33133		0.1215	0.17677	
Waist	-0.09384	1	-0.1292	0.48691		0.03765	1		0.2116	0.0058196	**	0.1799	0.039888	*

Phenotypes	Corr IL4RA	adj. P-value IL4RA	Corr STAT6	adj. P-value STAT6	Code STAT6	Corr SOCS1	adj. P-value SOCS1	Code SOCS1	Corr SOCS3	adj. P-value SOCS3	Code SOCS3
NEFA	0.1092	1	0.02005	1		0.2595	0.47868		0.2305	0.89508	
Cholesterol	-0.02213	1	-0.09164	0.063739	.	0.01553	1		0.03847	1	
LDL Cholesterol	0.0006553	1	-0.04502	1		0.02811	1		0.04604	1	
HbA1c	-0.04511	1	-0.05423	1		-0.08824	0.20059		-0.0003924	1	
Diabetes	0.005399	1	-0.04401	1		-0.04798	0.95616		0.01336	1	
HDL Cholesterol	-0.0119	1	-0.0142	1		-0.05246	1		-0.05923	1	
FPG	0.009098	1	-0.05436	0.63332		-0.005168	1		0.02636	1	
Age	0.04114	1	-0.01639	1		0.01739	1		-0.00813	1	
CrP	0.0235	1	-0.04186	1		-0.01027	1		0.009247	1	
Height	-0.001799	1	0.013	1		0.006587	1		0.005189	1	
WHR	0.0002111	1	-0.01756	1		0.04942	1		0.04963	1	
Body weight	-0.0179	1	0.03376	1		0.00606	1		0.01152	1	
BMI	-0.009851	1	0.03022	1		0.0116	1		0.01943	1	
Leptin	-0.02092	1	0.05424	1		-0.03097	1		0.07483	1	
Adiponectin	0.1011	1	0.271	0.063739	.	-0.155	1		-0.02871	1	
Body fat	-0.0483	1	0.1409	0.0037327	**	-0.07709	0.71984		-0.04153	1	
FPI	-0.05427	1	0.07514	1		-0.04117	1		-0.006601	1	
HOMA-IR	-0.01597	1	0.105	0.43108		-0.01946	1		0.00319	1	
Waist	-0.09384	1	0.04412	1		0.02423	1		0.2084	0.0071802	**

Correlation analysis of genes from human visveral adipose tissue samples with metabolic parameters (N=287). For correlation analysis a Pearson's correlation coefficient was used and adjusted for multiple comparisons using the Holm method**;** *p-value <0.05; **p-value <0.01; ***p-value <0.001

Table S3: Correlation values in subcutanous adipose tissue from genes with metabolic data

Phenotypes	Corr IL4	adj. P-value IL4	Code IL4	Corr IL13	adj. P-value IL13	Code IL13	Corr TIMP1	adj. P-value TIMP1	Code TIMP1	Corr FN1	adj. P-value FN1	Code FN1
Waist	0.09441	1		0.09075	1		0.06009	1		-0.09262	1	
CrP	0.0627	0.28587		0.05578	0.52016		-0.0222	1		-0.06598	0.19977	
WHR	0.05514	1		0.06689	1		0.06086	1		0.01719	1	
Diabetes	0.03706	1		-0.03238	1		0.02416	1		0.03374	1	
HbA1c	0.03296	1		0.008822	1		0.05109	1		0.03355	1	
FPG	0.02032	1		0.02396	1		0.01847	1		0.005457	1	
Leptin	0.01716	1		0.03092	1		0.04912	1		-0.04535	1	
Body fat	0.008673	1		0.06195	1		0.1437	0.0027585	**	0.08287	0.49371	
BMI	0.006404	1		0.03588	1		0.01892	1		-0.02649	1	
Body weight	-0.005308	1		0.02636	1		0.02326	1		-0.01921	1	
Height	-0.008995	1		-0.02387	1		-0.000252	1		-0.005138	1	
LDL Cholesterol	-0.01106	1		-0.07542	0.44013		0.007585	1		0.03457	1	
Age	-0.01693	1		-0.04278	1		0.006048	1		0.01736	1	
HDL Cholesterol	-0.01717	1		-0.02856	1		-0.0379	1		-0.01377	1	
HOMA-IR	-0.02094	1		0.02865	1		0.0701	1		0.05673	1	
FPI	-0.02294	1		0.02666	1		0.06393	1		0.05746	1	
Cholesterol	-0.03046	1		-0.07639	0.29111		-0.0105	1		0.01196	1	
NEFA	-0.06581	1		0.05777	1		0.1658	1		0.1366	1	
Adiponectin	-0.1818	0.95207		0.006323	1		0.1736	1		0.2213	0.32334	

Phenotypes	Corr LOX	adj. P-value LOX	Code LOX	Corr LOXL2	adj. P-value LOXL2	Code LOXL2	Corr CCN2	adj. P-value CCN2	Code CCN2	Corr TGFB1	adj. P-value TGFB1	Code TGFB1
Waist	-0.06845	1		0.2004	0.011471	*	0.06265	1		0.1273	0.52906	
CrP	-0.03066	1		-0.04694	0.89529		-0.01018	1		0.05267	0.61781	
WHR	-0.01296	1		0.1362	0.94542		0.008139	1		-0.01681	1	
Diabetes	-0.03185	1		-0.03266	1		0.0188	1		0.01563	1	
HbA1c	-0.01976	1		0.01721	1		-0.003748	1		0.04377	1	
FPG	-0.0442	1		-0.02286	1		-0.01662	1		0.03392	1	
Leptin	0.05995	1		0.1602	0.0024345	**	0.04428	1		0.03673	1	
Body fat	0.03192	1		0.1252	0.015915	*	-0.1656	0.00021952	***	0.2307	1.47×10^{-8}	***
BMI	-0.009559	1		0.04499	0.91601		-0.003537	1		0.03253	1	
Body weight	0.008256	1		0.05761	0.34449		0.008985	1		0.03018	1	
Height	0.0247	1		0.02698	1		0.02472	1		-0.02468	1	
LDL Cholesterol	0.09927	0.054517		0.05488	1		0.09779	0.059469		-0.07181	0.52906	
Age	0.002733	1		-0.01793	1		0.003754	1		-0.03841	1	
HDL Cholesterol	0.05506	1		0.002693	1		-0.0278	1		-0.01872	1	
HOMA-IR	-0.01505	1		0.1215	0.15579		-0.04436	1		0.02041	1	
FPI	0.01384	1		0.1496	0.015915	*	-0.02781	1		0.01033	1	
Cholesterol	0.09547	0.046051	*	0.02476	1		0.1249	0.0012666	**	-0.1006	0.025083	*
NEFA	0.1594	1		0.1383	1		0.2984	0.16541		0.0008025	1	
Adiponectin	0.1239	1		0.1289	1		-0.09528	1		-0.01139	1	

Phenotypes	Corr MMP3	adj. P-value MMP3	Code MMP3	Corr MMP9	adj. P-value MMP9	Code MMP9	Corr MMP12	adj. P-value MMP12	Code MMP12	Corr MMP13	adj. P-value MMP13	Code MMP13
Waist	0.04147	1		0.1079	0.81867		-0.0253	1		0.04177	1	
CrP	0.02421	1		0.001189	1		0.06568	0.18479		0.03251	1	
WHR	0.1355	1		0.1379	0.89648		0.009627	1		0.1573	0.79941	
Diabetes	0.02426	1		0.1038	0.00080252	***	0.0008792	1		-0.01316	1	
HbA1c	0.09018	0.16123		0.09005	0.13142		0.03738	1		0.02586	1	
FPG	0.04064	1		0.08099	0.04164	*	0.0138	1		0.01505	1	
Leptin	0.04366	1		-0.008564	1		0.03753	1		-0.0003729	1	
Body fat	0.005602	1		0.06617	0.89648		0.06535	1		-0.1175	0.036714	*
BMI	-0.001568	1		-0.001514	1		0.01118	1		0.007851	1	
Body weight	-0.00137	1		0.007407	1		0.009966	1		0.003721	1	
Height	-0.02006	1		0.005514	1		-0.001097	1		0.002995	1	
LDL Cholesterol	0.0171	1		0.03078	1		0.01203	1		-0.01414	1	
Age	-0.03292	1		-0.0167	1		-0.0293	1		-0.01701	1	
HDL Cholesterol	0.03506	1		-0.1342	0.00061833	***	-0.02068	1		-0.04186	1	
HOMA-IR	0.05361	1		0.1243	0.13142		0.09974	0.5347		0.04129	1	
FPI	0.04937	1		0.1245	0.095799	.	0.1031	0.3673		0.02746	1	
Cholesterol	0.03664	1		0.01996	1		-0.001061	1		-0.003705	1	
NEFA	0.2099	1		0.2188	0.81867		0.3829	0.014876	*	-0.03278	1	
Adiponectin	-0.3645	0.0012739	**	0.0008992	1		-0.3116	0.014065	*	-0.2083	0.47022	

Phenotypes	Corr MMP14	adj. P-value MMP14	Code MMP14	Corr COL1A1	dj. P-value COL1A	Code COL1A1	Corr COL3A1	adj. P-value COL3A1	Code COL3A1	Corr COL6A1	adj. P-value COL6A1	Code COL6A1
Waist	-0.278	3.27×10^{-5}	***	0.01097	1		-0.0639	1		-0.00455	1	
CrP	0.01132	1		-0.07939	0.035165	*	-0.06548	0.21114		-0.05361	0.64099	
WHR	-0.01853	1		-0.07409	1		-0.01652	1		-0.003216	1	
Diabetes	0.06741	0.11725		0.02507	1		-0.002823	1		0.02359	1	
HbA1c	0.04689	1		0.03358	1		-0.0002045	1		0.03255	1	
FPG	0.01207	1		0.014	1		-0.01602	1		0.009597	1	
Leptin	-0.1044	0.16696		0.01131	1		0.04246	1		-0.01101	1	
Body fat	-0.00506	1		0.1465	0.0020332	**	0.07519	0.76341		0.1091	0.076437	
BMI	-0.06747	0.11725		-0.01754	1		-0.01409	1		-0.01278	1	
Body weight	-0.04753	0.76531		-0.00824	1		-0.001229	1		-0.005783	1	
Height	0.01345	1		-0.007489	1		0.003345	1		-0.002873	1	
LDL Cholesterol	0.000324	1		0.008321	1		0.07775	0.3665		0.04677	1	
Age	0.029	1		0.01806	1		0.005963	1		0.01368	1	
HDL Cholesterol	0.03163	1		0.01484	1		0.0195	1		0.008201	1	
HOMA-IR	-0.1751	0.0035611	**	0.01425	1		0.04581	1		-0.0004362	1	
FPI	-0.1711	0.0027048	**	0.01544	1		0.0592	1		0.0112	1	
Cholesterol	-0.006444	1		-0.002093	1		0.05205	1		0.04401	1	
NEFA	-0.07008	1		-0.02023	1		0.1026	1		0.05959	1	
Adiponectin	0.3221	0.0075991	**	0.3331	0.0052731	**	0.2043	0.49692		0.2131	0.41135	

Phenotypes	Corr IL4RA	adj. P-value IL4RA	Code IL4RA	Corr IL13RA2	adj. P-value IL13RA2	Code IL13RA2	Corr IL13RA1	adj. P-value IL13RA1	Code IL13RA1	Corr JAK1	adj. P-value JAK1	Code JAK1
**Waist**	0.23	0.0016027	**	-0.01793	1		-0.1936	0.017604	*	-0.1898	0.023404	*
**CrP**	0.03946	1		-0.008146	1		-0.05646	0.45801		-0.04703	0.88804	
**WHR**	0.1321	1		0.021	1		-0.0226	1		0.01666	1	
**Diabetes**	0.06218	0.24928		0.01184	1		0.00234	1		-0.007771	1	
**HbA1c**	0.03681	1		0.06716	0.93253		-0.0596	1		-0.06198	0.88804	
**FPG**	0.05559	0.60427		0.02224	1		-0.0482	1		-0.07924	0.051667	
**Leptin**	0.04884	1		-0.02211	1		-0.1028	0.24313		-0.06179	1	
**Body fat**	-0.0634	1		0.05651	1		-0.04446	1		-0.09164	0.2203	
**BMI**	0.007464	1		-0.02139	1		-0.04508	1		-0.04174	1	
**Body weight**	0.00005764	1		-0.02202	1		-0.04211	1		-0.02425	1	
**Height**	-0.001156	1		-0.006053	1		0.009237	1		0.02551	1	
**LDL Cholesterol**	-0.06221	0.95383		0.03557	1		0.05076	1		0.1021	0.041291	*
**Age**	-0.02027	1		0.007806	1		0.05274	0.56539		0.03005	1	
**HDL Cholesterol**	-0.1112	0.010453	*	-0.01312	1		0.02991	1		0.04575	1	
**HOMA-IR**	0.03774	1		0.02137	1		-0.04914	1		-0.06539	1	
**FPI**	0.02434	1		0.002242	1		-0.03136	1		-0.03771	1	
**Cholesterol**	-0.04961	1		0.05015	1		0.02792	1		0.08898	0.075502	
**NEFA**	0.1185	1		0.1585	1		-0.1092	1		-0.08925	1	
**Adiponectin**	-0.0008344	1		-0.2837	0.042287	*	0.3389	0.0042981	**	0.2342	0.18223	

Phenotypes	Corr JAK2	adj. P-value JAK2	Code JAK2	Corr JAK3	adj. P-value JAK3	Code JAK3	Corr TYK2	adj. P-value TYK2	Code TYK2	Corr STAT1	adj. P-value STAT1	Code STAT1
**Waist**	-0.2051	0.0089634	**	0.2249	0.0023129	**	0.1468	0.21789		-0.1785	0.045569	*
**CrP**	-0.01586	1		0.04878	0.88025		-0.03734	1		-0.04117	1	
**WHR**	-0.0283	1		-0.02183	1		0.0297	1		-0.04413	1	
**Diabetes**	0.007907	1		-0.001971	1		-0.002256	1		0.008365	1	
**HbA1c**	0.01157	1		0.0128	1		-0.01565	1		-0.02868	1	
**FPG**	-0.0426	1		0.04184	1		-0.02332	1		-0.04336	1	
**Leptin**	-0.09804	0.31279		0.07456	1		-0.00901	1		-0.0246	1	
**Body fat**	-0.09942	0.14906		0.02026	1		0.07363	0.7879		-0.01188	1	
**BMI**	-0.05774	0.32029		0.03887	1		0.02646	1		-0.03242	1	
**Body weight**	-0.04377	1		0.01984	1		0.02469	1		-0.0139	1	
**Height**	0.02068	1		-0.02195	1		-0.00613	1		0.02291	1	
**LDL Cholesterol**	0.07806	0.31279		-0.08738	0.1548		-0.07233	0.49547		0.0651	0.88722	
**Age**	0.03404	1		-0.02559	1		-0.004658	1		0.02244	1	
**HDL Cholesterol**	0.05582	1		-0.0815	0.18954		-0.03752	1		0.02982	1	
**HOMA-IR**	-0.07394	1		0.04456	1		0.02514	1		-0.01951	1	
**FPI**	-0.05833	1		0.03502	1		0.02073	1		-0.001228	1	
**Cholesterol**	0.09105	0.068975		-0.09838	0.031935	*	-0.08148	0.17441		0.0545	1	
**NEFA**	0.1264	1		0.03903	1		-0.04987	1		0.05951	1	
**Adiponectin**	-0.1255	1		-0.1134	1		0.4249	4.67×10^{-5}	***	0.2135	0.40627	

Phenotypes	Corr STAT3	adj. P-value STAT3	Code STAT3	Corr STAT6	adj. P-value STAT6	Code STAT6	Corr SOCS1	adj. P-value SOCS1	Code SOCS1	Corr SOCS3	adj. P-value SOCS3	Code SOCS3
**Waist**	0.1625	0.11033		-0.1369	0.34538		0.08156	1		0.07787	1	
**CrP**	-0.01028	1		-0.03439	1		0.01929	1		0.02502	1	
**WHR**	0.06148	1		-0.01267	1		0.04089	1		0.09906	1	
**Diabetes**	0.009128	1		0.02764	1		0.0009642	1		0.05453	0.48571	
**HbA1c**	0.01728	1		0.0101	1		-0.01107	1		0.03783	1	
**FPG**	-0.03305	1		-0.009991	1		-0.03964	1		-0.004114	1	
**Leptin**	0.05658	1		-0.09971	0.31598		-0.002603	1		0.01128	1	
**Body fat**	-0.06022	1		-0.04424	1		-0.04721	1		-0.2538	2.2×10^{-10}	***
**BMI**	0.00798	1		-0.03554	1		-0.002206	1		-0.004315	1	
**Body weight**	0.009783	1		-0.03404	1		0.0105	1		0.0006594	1	
**Height**	0.01883	1		0.01029	1		0.002477	1		0.02755	1	
**LDL Cholesterol**	0.01993	1		-0.01287	1		0.001105	1		0.04617	1	
**Age**	-0.002127	1		0.029	1		-0.003018	1		0.002167	1	
**HDL Cholesterol**	-0.04852	1		-0.002972	1		0.004543	1		-0.08008	0.2145	
**HOMA-IR**	0.05714	1		-0.03742	1		-0.03002	1		-0.0206	1	
**FPI**	0.06572	1		-0.03195	1		-0.01994	1		-0.01409	1	
**Cholesterol**	0.008884	1		-0.03176	1		0.03542	1		0.08352	0.13613	
**NEFA**	0.03794	1		-0.2538	0.48447		0.4006	0.0084633	**	0.4715	0.00045831	***
**Adiponectin**	0.1637	1		0.3099	0.015073	*	-0.1238	1		-0.1934	0.54963	

Correlation analysis of genes from human subcutanous adipose tissue samples with metabolic parameters (N=287). For correlation analysis a Pearson's correlation coefficient was used and adjusted for multiple comparisons using the Holm method**;** *p-value <0.05; **p-value <0.01; ***p-value <0.001
